# Harnessing the sparing effect of FLASH-RT: From phenomenon observation, radiophysical determinants to molecular mechanisms and synergistic strategies

**DOI:** 10.1016/j.mtbio.2026.103487

**Published:** 2026-07-23

**Authors:** Zhihui Dou, Huiwen Lei, Jingfen Yang, MengYao Li, Shuting Wang, Xingting Bao, Li Ma, Jinjiang Li, Yong Yin, Tianyuan Dai

**Affiliations:** a Department of Radiation Oncology Physics and Technology, Shandong Cancer Hospital and Institute, Shandong First Medical University and Shandong Academy of Medical Sciences, Jinan, China; b Department of Oncology, Affiliated Hospital of Southwest Medical University, Luzhou, China; c Bio-Medical Research Center, Institute of Modern Physics, Chinese Academy of Sciences, Lanzhou, 730000, China; d Medical College of Northwest Minzu University, Lanzhou, China; e Department of Critical Care Medicine, The First Affiliated Hospital of Guangzhou Medical University, Guangzhou, 510120, China

**Keywords:** FLASH radiotherapy (FLASH-RT), FLASH effect, Ultra-high dose rate, Normal tissue sparing effect, Physical parameters, Clinical translation

## Abstract

FLASH radiotherapy (FLASH-RT) represents an innovative paradigm shift in radiation oncology. It is distinguished from conventional radiotherapy (CONV-RT) by the ultra-high dose rate (>40 Gy/s) radiation delivery to tumours within sub-millisecond bursts. Increasing preclinical studies have shown that FLASH-RT markedly reduces normal tissue toxicity without compromising antitumor efficacy, termed the "FLASH effect". Despite its potential, clinical implementation remains constrained by several challenges, including the incomplete understanding of its underlying biological mechanisms, the optimization of physical parameters, and ongoing technological hurdles. In this review, we systematically reviewed and synthesized the preclinical evidence across multiple organ systems, delineating the complex interplay of physical parameters that govern biological outcomes of FLASH-RT. Moving beyond phenomenological observations, this review provides a rigorous synthesis of mechanism ranging from initial physicochemical to biological processes, including transient oxygen depletion, radical recombination, differential DNA damage response, and mitochondrial reprogramming. A central focus is placed on the potent immunomodulatory role of FLASH-RT, highlighting its ability to preserve systemic immune competence and reshape the tumor immune microenvironment. These unique advantages have facilitated the initiation of FLASH-related clinical trials and position FLASH-RT as an exceptionally promising partner for next-generation immunotherapy, spatially fractionated approaches, and nanomedicine-based radio-sensitization research. While promising, mature clinical translation faces challenges. By synthesizing the interplay between ultra-high dose rate physics and molecular radiobiology, this review provides an integrated framework designed to translate fundamental FLASH effects into predictable and optimized therapeutic strategies for the next generation of precision cancer treatment.

## Graphical abstract

**Figure:**
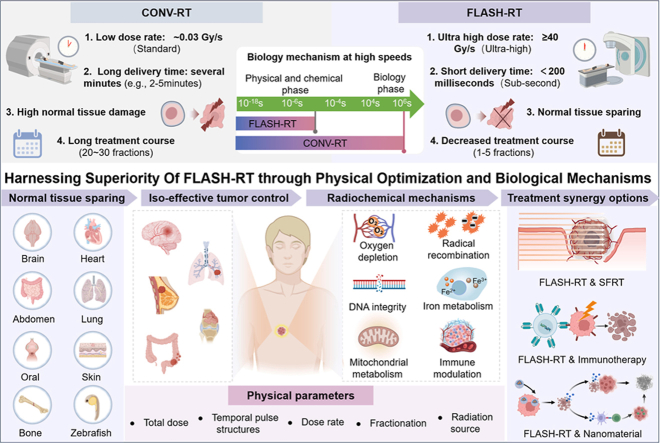

## 1 Introduction

Radiotherapy (RT) remains a cornerstone in the management of a wide variety of malignancies, either applied as a standalone treatment or integrated with other therapeutic modalities. An enduring objective in radiation oncology has been to improve the therapeutic ratio through innovations in physics, chemistry, and biology. In recent years, substantial progress has been made with the clinical implementation of image-guided radiotherapy (IGRT), adaptive radiotherapy (ART), and particle-based therapies [1,2]. Despite these technological advances in imaging, treatment planning, and dose delivery, progress in preferentially targeting tumours while sparing surrounding normal tissues has been incremental at best. Acute and long-term toxicities in healthy tissues continue to pose major clinical challenge, constraining dose escalation to tumour regions and compromising patients' quality of life.

**Fig. 1:**
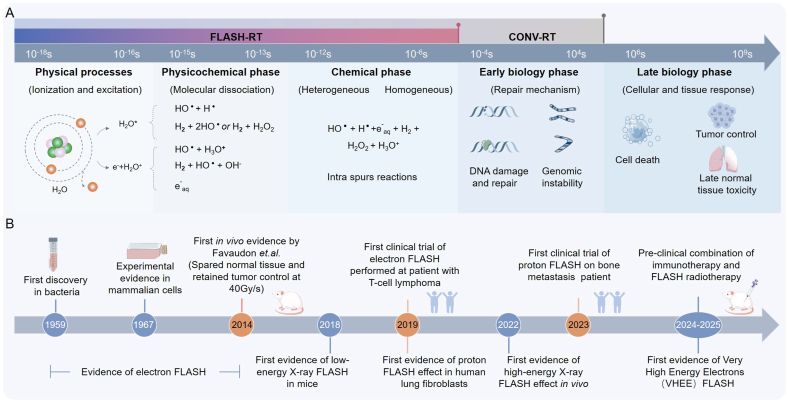
**A.** Illustration of the time scales of radiobiological events, involving physical processes, physicochemical phase, chemical phase, and biology phase. **B.** The timeline of advances in FLASH irradiation.

One revolutionary strategy aimed at healthy tissue preservation is ultra-high dose rate (UHDR) irradiation, known as FLASH radiotherapy (FLASH-RT). FLASH-RT delivers radiation at dose rates of ≥40 Gy/s within milliseconds*,* which is several thousand times higher than the dose rates of 0.03–0.05 Gy/s used in conventional radiotherapy (CONV-RT) (Fig. 1) [3,4]. An growing body of preclinical evidence has demonstrated that FLASH-RT can markedly reduce normal tissue toxicities without compromising tumour control when compared with CONV-RT (Fig. 1) [2]. This differential effect on normal tissues and tumours referred to as the “FLASH effect”, introduces a fundamentally new therapeutic paradigm with the potential to overcome dose-limiting toxicities that constrain conventional radiotherapy [5,6]. Nevertheless, despite its considerable promise, the clinical translation of FLASH-RT remains hindered by an incomplete understanding of its biological mechanisms and the lack of a well-established relationship between key physical parameters and biological outcomes.

**Fig. 2:**
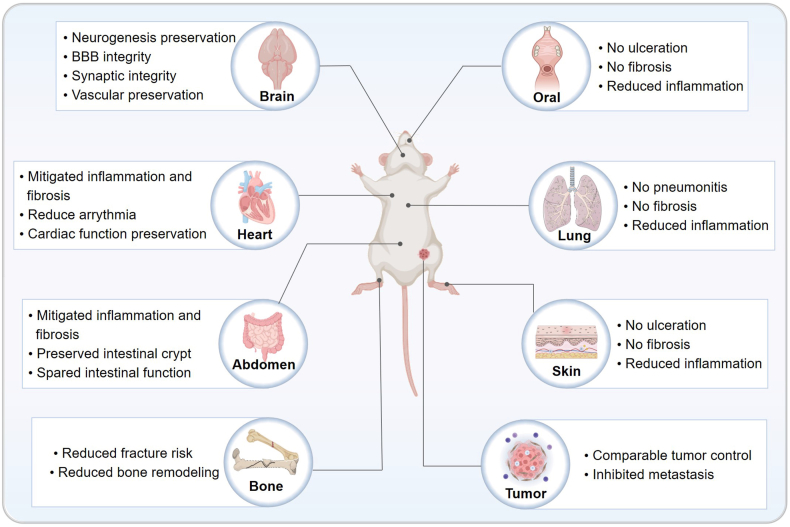
**FLASH-RT reduced normal tissue injury across multiple organs.** CONV-RT induces extensive normal tissue injury characterized by elevated cell death, inflammatory and fibrotic responses across multiple organs. In contrast, ultra-high dose rate FLASH-RT alters radiation-induced cell fate by attenuating cellular stress and inflammation, thereby preserving tissue architecture and regenerative capacity in the skin, brain, lung, and gastrointestinal tract. These effects are accompanied by reduced senescence-associated and fibrotic remodeling, suggesting improved long-term tissue preservation. Importantly, FLASH-RT maintains comparable tumor cell killing, highlighting its ability to differentially modulate cell death pathways in normal versus tumor tissues.

Herein, we reviewed the accumulating experimental evidence across multiple organ systems, which indicated that the radioprotective effects of FLASH-RT may extend beyond localized tissues and exhibit systemic characteristics (Fig. 2). However, the tissue-specific parameters and conditions required to reliably elicit the FLASH effect remain to be fully elucidated. We further discussed the key physical parameters considered essential for the reproducible induction of FLASH effect. In parallel, the prevailing physicochemical hypotheses alongside emerging biological mechanisms were critically evaluated, highlighting that neither framework alone sufficiently accounts for the complexity of the FLASH phenomenon. Finally, we summarized current clinical progress and major translational challenges in FLASH-RT, and outline prospective combinatorial strategies, including integration with immunotherapy, nanoparticle-mediated targeted drug delivery, and spatiotemporal dose modulation. These approaches may offer promising avenues toward achieving more effective and less toxic radiotherapy.

## 2 Systemic organ and tissue sparing of FLASH-RT

### 2.1 Skin tissue

Radiation dermatitis (RD) is one of the most common and dose-limiting toxicities of radiotherapy, affecting nearly 95% of patients and significantly impairing quality of life [7]. Despite its high incidence, effective preventive strategies remain limited, and no standardized management guidelines have been established. Current clinical practice primarily relies on minimizing skin dose and implementing supportive care measures [8]. FLASH-RT has consistently demonstrated pronounced skin-sparing effects across a wide range of preclinical models, including cats, dogs, pigs, and particularly murine systems. At defined time point, FLASH irradiation significantly attenuated skin toxicities such as epilation, ulceration, necrosis, and fibrosis [[9], [10], [11]]. These studies have employed diverse irradiation parameters, with dose rates typically ranging from ∼40 to 250 Gy/s and single-fraction doses from 15 to 65.9 Gy, predominantly delivered via electron, photon or proton beams.

Recent research highlights the dose-rate dependency of acute skin toxicity, revealing that the incidence of severe lesions diminishes as the dose rate increases. Notably, these findings demonstrated a FLASH effect for the current endpoint can be seen at dose rates as low as <1 Gy/s, a threshold significantly lower than the ∼40 Gy/s previously established. This underscores the critical importance of the reference dose rate of CONV in FLASH studies [12]. It also indicates that, depending on the dosage and the specific endpoints adopted, even a slight increase in dose rate can significantly reduce severe toxicity. For clinical applications and the optimization of FLASH-RT protocols, they suggested that gradually increasing the dose rate from low to moderate levels may be more advantageous than drastically increasing it to high levels [12]. Furthermore, the efficacy of the FLASH effect is sensitive to temporal delivery patterns. The use of field repainting in proton FLASH-RT consistently exacerbates toxicity, thereby necessitating higher instantaneous dose rates to maintain the same degree of skin sparing [12]. Regarding the dose-response relationship, using depilation and fibronecrosis as endpoints, Vozenin et al. reported that 25 Gy delivering at conventional dose rate induced skin toxicity comparable to that observed with 34 Gy administered via FLASH-RT in a porcine model, corresponding to a dose-modifying factor (DMF) of at least 1.3 for single-fraction FLASH irradiation [10,13]. Similarly, murine studies showed that electron FLASH-RT significantly reduced both the incidence and severity of skin ulceration at doses of 30 and 40 Gy compared with CONV-RT at 8 weeks post irradiation [14]. Kristensen et al. further demonstrated clearly separated dose-response curves(19.4–57.6 Gy) and quantitatively determined DFMs ranging from 1.45 to 1.54 for acute damage and approximately 1.15 for late fibrotic injury following FLASH-RT [15]. Similarly, Paillas et al. highlighted a significant skin-sparing effect of FLASH-RT on the onset and severity of radiotoxicity, reporting a DMF of approximately 1.5[16]. Proton FLASH-RT also demonstrated comparable protective effects. Within the spread-out Bragg peak (SOBP), a mean protection ratio of 1.40 (range: 1.35–1.46) was reported for acute toxicity, indicating that a 35–46% higher dose is required to elicit equivalent acute toxicity relative to CONV-RT, and approximately 18% higher dose for late effects [17]. Further comparisons between entrance and SOBP regions suggest that the skin-sparing effect of proton FLASH is largely independent of treatment depth [18].

Notably, splitting the total dose into smaller deliveries with the mean duration of the pauses was 121 to 126 s, simulating the effect of multiple overlapping fields, compromised the FLASH effect to different degrees across the included toxicity level endpoints of skin [12]. A loss of FLASH sparing was also observed when the total dose was split into a dual pulse delivery or introduce beam interruption time ≥15s [19,20]. However, FLASH skin-sparing effect is maintained for irradiation fractionated doses of 3 × 10 Gy or 3 × 15 Gy delivered in 3 consecutive days [16]. These findings indicated that the FLASH effect is governed by a critical temporal window. While high instantaneous dose rates are required to mitigate the loss of the FLASH effect during protracted delivery (such as multi-field sequencing), these must be reconciled with the limitations of single-field dose uniformity. This presents a formidable challenge for clinical implementation, necessitating an optimization strategy that reconciles the high-dose-rate requirements for FLASH-induced protection with the clinical necessity for precise spatial dose delivery.

### 2.2 Brain and neurocognition

Cranial radiation therapy (CRT) remains a cornerstone in the management of primary and metastatic brain tumours but is frequently associated with substantial acute and long-term toxicities [21,22]. CRT induces damage to both white and grey matter through inflammation, angiogenesis and cell death, resulting in neuronal loss, impaired neurogenesis, and persistent cognitive deficits [[23], [24], [25]]. Although strategies to mitigate neurotoxicity, such as hippocampal-avoidance whole-brain radiotherapy, stereotactic radiosurgery, and pharmacological interventions including memantine, have demonstrated modest clinical benefits [26], there remains a critical unmet need for effective approaches to prevent radiation-induced brain pathophysiological alterations and cognitive decline [27], especially pediatric patients.

FLASH-RT has emerged as a transformative strategy for mitigating radiation-induced brain injury (RIBI). Pioneering work by Montay-Gruel et al. demonstrated that FLASH-RT conferred superior neurocognitive preservation in both healthy and glioblastoma-bearing mice compared with CONV-RT, as assessed by a comprehensive battery of behavioural assays at both short (1 month) and long term (6 month) endpoints [4,28,29]. In radiosensitive juvenile mouse models, both single-dose (8 Gy) and hypo-fractionated (2 × 10 Gy) FLASH-RT regimens more effectively preserved cognitive performance and electrophysiological properties than CONV-RT [30,31]. Equivalent tumor control for FLASH and conventional RT was also demonstrated in DIPG orthotopic model, a universally fatal pediatric brain tumor [32]. These benefits have been consistently linked to attenuation of neuroinflammation mediated by diminished reactive oxygen species (ROS) production, as evidenced by diminished microglial activation, reduced astrogliosis, and downregulation of pro-inflammatory cytokines [[28], [29], [30], [31],^33^]. In addition, FLASH-RT has been shown to preserve hippocampal neurogenesis, synaptic integrity, dendritic spines, and microvasculature of blood-brain barrier (BBB) [28,31,33]. Furthermore, FLASH-RT achieves comparable tumour control efficacy to CONV-RT in orthotopic glioblastoma patient-derived xenograft models while sparing normal brain tissue, thereby supporting the dual therapeutic advantage of FLASH-RT [34].

Most studies investigating the neuroprotection effect of FLASH-RT employed single whole brain dose of 8-14 Gy, with consistent evidence indicating that the FLASH effect was absent in cohorts exposed to 14 Gy [4,28]. Recent studies examining fractionation strategies demonstrated that hypo-fractionated FLASH-RT regimens (2☓7 Gy and 3☓10 Gy) prevented neurocognitive decline relative to CONV-RT, supporting hypofractionation as a potentially favorable approach for maximizing FLASH benefits in clinical practice [4,28,35]. Importantly, FLASH-RT remains equally effective as CONV-RT in delaying tumour growth across both single-dose and fractionated schedules [4]. Nevertheless, a key limitation of the current literature lies in the discrepancy between preclinical experimental designs and clinical radiotherapy paradigms. Whereas most clinical glioma protocols rely on prolonged multifractionated regimens (10☓3 Gy), many preclinical FLASH studies compare single high-dose FLASH-RT with equally high-dose CONV-RT controls, which may exaggerate toxicity in the CONV group. Future studies should incorporate multifractionated CONV-RT control arms to enable more rigorous and clinically meaningful comparisons. These findings provide compelling preclinical and mechanistic insight into the potential of FLASH-RT to mitigate long-term neurocognitive sequelae, particularly in pediatric and long-term brain tumor survivors. Nevertheless, some negative results should not be overlooked. A recent study of clinically relevant brain sub-volume irradiation demonstrated no neuroprotective effect six-months post-irradiation. However, analysis of skin reaction suggests a trend towards a protective FLASH effect [36]. This discrepancy indicates that partial brain irradiation may trigger distinct molecular, cellular, and neurocognitive responses that consequently impact the FLASH effect. Therefore, future studies should not only map dose-response relationships over diverse endpoints and time scales but also elucidate how variations in irradiated volume influence the overall efficacy of FLASH-RT.

### 2.3 Radiation-induced lung injury (RILI)

The management of RILI has long represented a persistent challenge in optimizing radiotherapy for thoracic malignancies. Seminal work by Favaudon et al. first demonstrated that FLASH-RT not only achieved effective tumor eradication but also markedly reduced the incidence and severity of complications following bilateral thoracic irradiation [37]. Specifically, while CONV-RT delivered at 15 Gy triggered pulmonary fibrosis in all mice through activation of the TGF-β/SMAD signalling cascade, no such damage was observed following FLASH-RT at doses up to 20 Gy over 36 weeks of follow-up [37]. FLASH-RT protected smooth muscle and epithelial cells of vascular and bronchi from acute radiation-induced apoptosis, an effect partially reversed by TNF-α pretreatment [37]. In addition, FLASH irradiation was shown to reduce DNA damage in normal cells, preserve lung progenitor cells and reduce replicative senescence [38]. Subsequent studies using proton FLASH delivered on clinical platforms corroborated this protective phenotype, showing up to a 30% reduction in lung fibrosis and improved survival compared with CONV-RT. In addition, proton FLASH-RT at ablative dose levels (>60 Gy) also conferred localized protection against radiation-induced lung and skin injury in a preclinical setting [39]. In terms of physical parameters, an average dose rate exceeding 40 Gy/s was sufficient to elicit lungs protection [37]. Gao et al. further reported that FLASH-RT delivered at 250 Gy/s was more effective than 100 Gy/s in improving survival and preserving alveolar morphology. With respect to dose fractionation, a 30 Gy regimen delivered in two fractions was more detrimental to lung structure and survival than either single-fraction or four-fraction schedules [40]. Importantly, a threshold dose of 2 Gy per fraction has been established for eliciting the sparing effect in lung models, providing a critical reference for clinical translation [41,42]. Despite consistent evidence that the protective effects of FLASH-RT against pulmonary fibrosis and inflammatory cell infiltration both at single- and multi-fraction thoracic radiation [[42], [43], [44]], the uniformity of FLASH protection remains a subject of debate. Notably, recent study examining the FLASH effect at early time points reported comparable acute lung injury following FLASH-RT and CONV-RT, with no significant differences in fibrosis markers or pro-inflammatory cytokine expression [45]. Overall, the existing body of evidence underscores the multifaceted protective potential of FLASH-RT against RILI, mediated by the integrated regulation of DNA damage responses, immune cell dynamics, and chronic fibrotic pathways. However, the observed discordance between early-stage acute inflammatory responses and long-term fibrotic outcomes highlights a critical gap that the "FLASH-sparing" effect may follow a temporal trajectory that is not immediately reflected in transient inflammatory markers.

### 2.4 Gastrointestinal tract

Radiotherapy remains one of the most effective cytotoxic modalities for the treatment of abdominal and pelvic malignancies. However, its curative potential is constrained by the pronounced radiosensitivity of the gastrointestinal tract. Approximately 60% to 80% of patients experience acute bowel toxicity during or after treatment. Preclinical studies have demonstrated that FLASH-RT can substantially preserve intestinal structure and function following irradiation. In murine models, a single high dose of (12∼16 Gy) total abdominal irradiation (TAI) delivered at FLASH dose rate significantly reduced lethality from gastrointestinal syndrome and spare intestinal stem cell (ISC) essential for crypt regeneration compared with CONV-RT(46). Further study revealed that the improved morbidity after proton FLASH-RT were associated with greater proliferation of damage-induced epithelial progenitor cells dependent on IFN-I signaling [46]. Importantly, abdominal FLASH-RT achieved equivalent tumor control as CONV-RT while markedly reducing intestinal toxicity in a preclinical mouse model of ovarian cancer metastasis [47]. Shi et al. further demonstrated that FLASH X-ray irradiation reduced intestinal pyroptosis, thereby counteracting CONV-RT–induced gastrointestinal toxicity in the context of PD-L1 blockade, while maintaining comparable efficacy in both primary and abscopal tumour control [48]. The intestinal sparing effects of 6 MV X-ray FLASH were also confirmed by Zhu et al. in BALB/c nude mice [49]. Nevertheless, caution is warranted, as the intestinal FLASH effect appears to be highly dependent on physical beam parameters. Venkatesulu et al. reported that ultra-high dose rate electron irradiation at 35 Gy/s exacerbated gastrointestinal mucosal injury and mortality compared with CONV-RT [50]. Similarly, studies using pencil beam scanned proton FLASH showed reduced survival following 14–18 Gy FLASH-RT relative to CONV-RT, with no significant differences in crypt regeneration or fibrosis [51,52]. These findings were consistent with the latest comparisons between synchrotron-based proton FLASH and linac-based electron FLASH at matched mean dose rates (MDR). Mice received proton FLASH SOBP had the lowest survival rates and regenerating jejunal crypts, serving to caution that abdominal proton FLASH may increase acute gastrointestinal toxicity rather than mitigate it [53]. However, a recent study has reported positive findings, demonstrating preserved gut commensal microbiomes, intestinal stem cells and improved survival rates at a dose of 14–18 Gy delivered at 50 Gy/s [54]. Intestinal tissue is highly sensitive to radiation, even minute differences during the irradiation process can lead to divergent outcomes. However, subtle variations in the size of the radiation field alone seem insufficient to explain these contradictory results; A more detailed disclosure of machine and procedural parameters is required to elucidate the underlying causes. The variations in dose per pulse (DPP) and MDR strongly influenced the magnitude of intestinal sparing, further emphasizing the sensitivity of the FLASH effect to beam configuration [55,56]. In summary, while FLASH-RT holds promise for expanding the therapeutic window in abdominal radiotherapy, careful characterization of the physical and biological determinants is essential to ensure safe and effective clinical translation.

### 2.5 Others tissues

The normal tissue sparing effects of FLASH-RT have also been demonstrated in several other organ systems. In orthotopic murine models of head and neck cancer, both single dose and fractionated proton FLASH-RT significantly improved survival compared with CONV-RT by reducing salivary gland dysfunction and oral mucositis while maintaining tumour control [57]. In addition, both proton and photon FLASH has been shown to attenuate cardiac inflammatory and profibrotic responses, thereby reducing myocardial fibrosis and preserving radiation-induced changes in cardiac function, remodeling and arrythmia in preclinical models, providing a rationale for its evaluation in thoracic malignancies [58,59]. Skeletal tissues similarly benefit from FLASH irradiation. Compared with standard proton radiotherapy, bones treated with proton FLASH-RT exhibited reduced damage, including diminished bone resorption and fewer activated osteoclast and osteoblast clusters. In a murine tibial reirradiation model, Verginadis et al. reported fractures in 83.3% of mice receiving hypo-fractionated conventional proton reirradiation, compared with only 20.0% in those re-irradiated with proton FLASH-RT, indicating a substantial reduction in chronic bone toxicity [60].

Collectively, FLASH-RT confers broad normal tissue protection across multiple organ systems, including the brain, head and neck region, thorax, abdomen, and skeletal system, while preserving antitumor efficacy. However, several preclinical studies have reported neutral or adverse outcomes (Table 1). These studies employed heterogeneous radiation modalities, dose, fractionation schemes, as well as toxicity endpoints, complicating the identification of consistent determinants of FLASH efficacy. Systematic integration of both positive and negative data will therefore be essential to delineate the operational parameter space of FLASH-RT and to guide its rational and safe clinical implementation.

**Table 1:** The negative outcomes of FLASH-RT in preclinical research.

Study	Modality	System	Dose rate	Dose/Fraction	Main findings
Liu K [53].	Proton	Abdomen	150 Gy/s (S-T) and 230 Gy/s, (SOBP)	12-14 Gy/1F	Lowest survival rates and crypts regeneration with FLASH;
Bell [51].	Proton	Abdomen	150 Gy/s	14 Gy/1F	Significantly impaired survival after abdominal proton FLASH;
Zhang QX [52]	Proton	Partial abdomen	120 Gy/s	16-18 Gy/1F	Worse survival in FLASH; No spare of intestine and lymphocyte;
Venkatesulu BP [50]	Electrons	Abdomen	35 Gy/s	16 Gy/1F	FLASH-RT is more potent in causing gastrointestinal mucosal toxicity;
	Electrons	KPC, Panc02	35 Gy/s	0-8 Gy/1F	FLASH-RT is more potent in reducing clonogenicity of cells;
	Electrons	Spleen Heart	35 Gy/s	5 Gy/1F	FLASH-RT is more potent in depleting immune cells;
				2 Gy/5F	
				8 Gy/1F	
Beyreuther.E [61]	Electrons	Zebrafish embryo	100 Gy/s	0-43 Gy/1F	No survival difference;
Smyth LML [62]	X-ray	Total body	41 Gy/s	3.9-8.4 Gy	No evidence of normal tissue sparing relative to CONV-RT;
		Head	41 Gy/s	7.6-14.5 Gy	
		Abdomen	41 Gy/s	9.2-17.2 Gy	
Cuitiño MC [63]	Electrons	Abdomen/pelvis	>100 Gy/s	8 or 16 Gy/1F	Comparable short-term effects on gonads;

## 3 The key physical determinants that modulate radiobiological responses of FLASH-RT

FLASH-RT is commonly defined by the delivery of single doses ≥10 Gy at a mean dose-rate exceeding 40 Gy/s, typically within microsecond-scale irradiation times [4,37,64]. However, accumulating evidence indicates that FLASH effect cannot be sufficiently described by a single parameter. Instead, it represents a complex and multi-dimensional phenomenon governed by the interplay among several physical variables, including total or fraction dose, instantaneous dose rate (IDR), MDR, DPP, the temporal structure of the beam (number and width of pulses), and radiation quality (electrons, photons, protons and heavy ions) [65]. To date, most experimental demonstrations of the FLASH effect have relied on simplified irradiation paradigms, frequently involving single high-dose exposures delivered from a single beam direction under highly controlled conditions. While these approaches have been instrumental in establishing proof-of-concept, they only partially reflect the complexity of modern radiotherapy standards, which requires precise dose conformity, fractionation, and robust reproducibility across heterogeneous tissues and tumour types [66,67]. In this context, a systematic evaluation of how individual physical parameters and their interactions shape biological responses is essential. Such an analysis not only provides a mechanistic framework to reconcile divergent experimental findings but also establishes a rational foundation for optimizing FLASH-RT protocols that are both biologically effective and clinically translatable.

### 3.1 Dose rate thresholds and total irradiation time

**Fig. 3:**
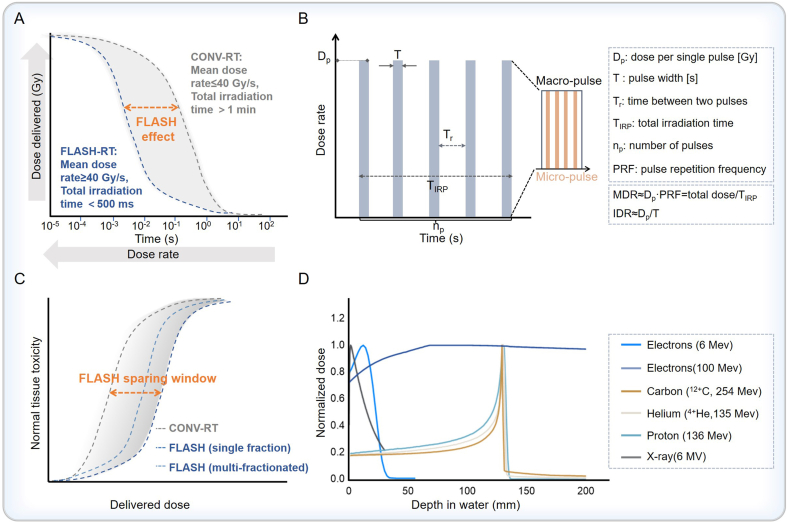
**Conceptual framework and physical characteristics of FLASH-RT. (A)** Schematic comparison of dose delivery kinetics between CONV-RT and ultra-high dose rate FLASH radiotherapy. FLASH-RT is characterized by extremely high mean dose rates (≥40 Gy s^−1^) delivered within sub-second time scales (typically <500 ms) in contrast to CONV-RT, which delivers dose over minutes at much lower dose rates. The shaded region indicates the putative “FLASH effect,” i.e., enhanced normal tissue sparing at comparable tumor dose. **(B)** Temporal structure of FLASH-RT. Key parameters defining dose delivery are illustrated, including dose per pulse (D_p_), pulse width (T), pulse repetition frequency (PRF), number of pulses (n_p_), time between pulses (Tᵣ), and total irradiation time (T_IRP_). Both macro-pulse and micro-pulse structures are shown, highlighting that the mean dose rate (MDR = D_p_ × PRF) and instantaneous dose rate (IDR ≈ D_p_/T) jointly determine FLASH conditions. **(C)** Dose–response relationship for normal tissue toxicity under fractionated FLASH-RT. Increasing fractionation progressively attenuates this protective effect, resulting in convergence toward the conventional dose–response relationship. **(D)** Depth–dose distributions in water for different radiation modalities relevant to FLASH-RT, including electrons, protons, helium ions, carbon ions, and conventional X-rays. Illustrating the effect of beam quality on FLASH irradiation.

Dose rate is fundamental parameter distinguishing FLASH from CONV. Research spans from complex biological models to simple chemical systems. Early and systematic dose rate de-escalation studies have consistently demonstrated that normal tissue sparing emerges only above a critical MDR threshold (Fig. 3A). Montay-Gruel et al. delivered 10 Gy to the mouse brain using a single 1.8 μs pulse (5.6 × 10^6^ Gy/s) and progressively reduced the dose rate down to 0.1–500 Gy/s by increasing pulse number. Neurocognitive function is fully preserved at rates above 100 Gy/s, but gradually deteriorated with decreasing dose rate, and was completely disappeared below approximately 30 Gy/s [68]. Similar dose rate–dependent sparing effects were observed in zebrafish embryos, where increasing MDR robustly enhanced tissue protection [50,69]. At the cellular level, alkaline comet assays revealed that DNA damage in peripheral blood lymphocytes irradiated across a wide range of dose rates (0.1–2 kGy/s) became significantly reduced only when dose rates exceeded ∼30 Gy/s [70]. The mechanism underlying FLASH spare normal tissues is linked to suppressed ROS production [71]. Measurement of H_2_O_2_ production in cell-free systems, including aqueous solutions and 4% albumin in water, demonstrated that the radiolytic H_2_O_2_ yields decrease as MDR increases from 0.14 to 1500 Gy/s [28,71]. Importantly, this dose rate–dependent reduction in ROS generation has been observed for both low linear energy transfer (LET) photon beams and high-LET carbon ion irradiation, underscoring the generality of this phenomenon across radiation qualities [72].

Considering the negative results observed at dose rates of 35-41 Gy/s [50,62], an MDR exceeding ∼40 Gy/s is commonly regarded as a necessary threshold for eliciting FLASH-associated normal tissue sparing. However, this threshold is neither universal nor sufficient on its own. Multiple studies reporting absent or even detrimental FLASH effects at dose rates of >40 Gy/s highlight the importance of additional modulating parameters. Tissue type and radiation modality critically influence the effective dose rate window. For example, intestinal crypt damage was significantly reduced when 7.5–12.5 Gy electron irradiation was delivered at an MDR of 280 Gy/s [56], whereas proton irradiation at 14–18 Gy and an MDR of 120 Gy/s failed to induce a protective effect in the same tissue [52]. These findings indicate that dose rate thresholds might be particle- and tissue-specific, tailored optimization rather than universal dose rate prescriptions is necessary.

Although FLASH-RT is commonly defined by a high MDR, recent studies have demonstrated that the FLASH effect arises from the interaction between MDR and DPP. MDR has been identified as more predictive of FLASH outcomes than IDR in several models [[73], [74], [75]]. Cao et al. also showed that hydrated electrons during electron FLASH increased with increasing instantaneous dose rates (0.18–0.33 MGy s^−1^), which may be attributed to the greater energy density delivered during each pulse [76], while a different trend for 230 MeV protons [77]. Further evaluation of interaction between MDR and DPP showed that at a fixed high MDR (>100 Gy/s), increasing DPP enhanced gastrointestinal tissue sparing in mouse model [55]. Conversely, at a fixed high DPP, similar survival benefits were observed across a broad range of MDRs, with tumour control remaining unaffected [55]. Collectively, these data indicated that the sparing effect of FLASH-RT depends on the interplay between MDR and DPP. While preclinical evidence suggested uncompromising tumour control efficacy by dose rate variations, this conclusion remains to be validated across a broader spectrum of tumour types and microenvironmental contexts [37]. For successful clinical translation, future research must focus on establishing tissue-specific dose rate thresholds that integrate radiation type, DPP, and MDR, accounting for the profound variations in radiosensitivity and energy deposition.

### 3.2 Temporal pulse structures

Modern radiation delivery systems inherently operate in a pulsed mode, generating complex temporal beam structures that extend beyond simple dose rate metrics. In the context of FLASH-RT, irradiation time is extremely short typically less than 100 ms and often within a few microseconds, which rendering temporal pulse characteristics a critical determinant of biological outcome of FLASH-RT [78]. These characteristics include pulse width, number of pulses, pulse repetition frequency, time between two pulses, and macro-versus micro-pulsing patterns, all of which influence energy deposition kinetics and subsequent radiochemical reactions (Fig. 3B). Experimental evidence increasingly indicates that temporal pulse structure can substantially modulate the magnitude of the FLASH effect. In a murine whole-abdomen irradiation model, Ruan et al. demonstrated that increasing the number of pulses from a single delivery to multiple pulses, or prolonging the inter-pulse interval in dual-pulse schemes, progressively reduced intestinal crypt survival following FLASH-RT, indicating that extending the effective irradiation time might compromise the protective effect [56]. These observations are concordant with the studies by Karsch et al. who reported that the macro pulsing induced prolongation of treatment time regime reduced the protecting effect compared to the maximum regime delivered at same bunch but higher mean dose rate [73]. Similarly, in proton FLASH-RT, delivery of the total dose within a single macro-pulse was shown to significantly reduce normal tissue injury, whereas splitting the same dose into multiple macro-pulses separated by a 2-min interval markedly diminished or completely abrogated the sparing effect [79]. Collectively, these results emphasize that maintaining temporal compactness of dose delivery is essential for preserving FLASH-induced normal tissue protection. Intriguingly, however, studies based on photon FLASH showed that both single pulse and multiple macro-pulse (with an interval of 1 min) can reduce radiation-induced pulmonary pathology in mice [41]. This discrepancy suggests that differences in radiation quality, pulse microstructure, or radiochemical kinetics may alter the sensitivity of biological systems to temporal dose dispersion. Taken together, temporal pulse structure represents an independent and non-negligible physical dimension of FLASH-RT, distinct from but tightly coupled to dose rate metrics. Parameters such as pulse number, pulse width, repetition frequency, and inter-pulse spacing collectively define the effective irradiation time and might influence the balance between radical production, recombination, and biological damage fixation [75,80].

### 3.3 Fractionation strategy

Fractionated radiotherapy constitutes the clinical standard of care for vast majority of solid tumours, enabling normal tissue repair while maintaining tumour control through differential radiosensitivity. In contrast, majority of preclinical FLASH-RT studies have predominantly focused on single-fraction or ultra-hypofractionated regimens, leaving the biological outcomes of clinically relevant fractionation schedules incompletely characterized. Understanding how fractionation modulates the FLASH effect is therefore essential for bridging the gap between experimental observations and clinical implementation (Table 2). Early study in whole-brain irradiation models consistently demonstrated that a single dose of 8-12 Gy delivered at FLASH rates with protons, electrons or X-rays consistently alleviated radiation induced brain injury, including memory deficits, hippocampal damage, and reactive astrogliosis, however, this sparing effect was lost at a single fraction dose of 14 Gy [[28], [29], [30],^68^,^81^]. Extending beyond single-fraction paradigms, multiple studies have examined hypo-fractionated FLASH-RT regimens. Montay-Gruel and colleagues demonstrated that hypo-fractionated FLASH-RT of two to three fractions (2 × 10 Gy, 3 × 10 Gy, or 2 × 7 Gy) conferred significant and long-term neuroprotection compared to CONV-RT, while maintaining equivalent efficacy in delaying glioblastoma growth [4,31,35]. Although a regimen with a lower dose per fraction (4 × 3.5 Gy) failed to elicit a detectable FLASH benefit [4], their subsequent work revealed that a standard clinical fractionation schedule (10 × 3 Gy) delivered at FLASH dose rate preserved long-term potentiation (LTP) [82], suggesting the existence of a cumulative dose threshold for the FLASH effect in low dose-per-fraction settings. The benefits of FLASH-RT in split-dose have also been extended to re-irradiation scenarios. Compared with standard proton irradiation, both a single 12 Gy dose and a hypofractionation scheme (3 × 6.4 Gy) demonstrated a protective effect on intestinal tissues following reirradiation. Similarly, in the murine leg model, hypofractionated FLASH reirradiation (3 × 11 Gy) markedly reduced skin dermatitis, lymphedema and tibial fractures compared to CONV-RT(60).

**Table 2:** The effect of hypo-fractionated FLASH-RT regiment in preclinical research.

FLASH modality	System	Fraction regime	Time scale	Normal tissue sparing	Tumor control	Ref
Electrons	H454 glioma bearing animals and human GBM (U87) animals	2 × 7 Gy	Separated by 24 h	Excellent performance than CONV	Similar	(4)
		4 ☓ 3.5 Gy	Separated by 24 h	No particular benefit of FLASH-RT	Similar	(4)
		3 ☓ 10 Gy	Separated by 48 h	Excellent performance than CONV-RT	Similar	(4)
Electrons	Juvenile mice brain	2 ☓ 10 Gy	Separated by 48 h	Significant and long-term normal tissue protection	/	(32)
Electrons	Whole brain	3 ☓ 10 Gy	Separated by 48 h	Functional preservation of cognition and LTP	/	(35)
Electrons	Heart	5 ☓2 Gy	Separated by 24 h	No sparing effect even more severe	/	(50)
Proton	Intestine	3 ☓ 6.4 Gy	Separated by 24 h	Decrease fibrosis and enhance survival	/	(60)
Proton	Skin	3 ☓ 11 Gy	Separated by 24 h	Spared dermatitis and lymphedema	/	(60)
Proton	Bone	3 ☓ 11 Gy	Separated by 24 h	Reduces bone fracture	/	(60)
Electrons	Whole brain	10 ☓ 3 Gy	Monday to Friday	Preservation of LTP	/	(82)
Electrons	Whole-abdominal	2 ☓8-13 Gy	Separated by 24 h	Attenuated FLASH sparing effect	/	(83)
Electrons	Whole-abdominal	10 ☓5-7 Gy	Monday to Friday across two weeks	Loss of FLASH sparing effect	/	(83)

Despite these encouraging results in late-responding tissues and re-irradiation contexts, the reproducibility of this effect in acute-responding tissues under fractionated protocols has been inconsistent. Recent study of whole abdominal irradiation reported that FLASH effect was reduced with two fractions (2×8-13 Gy) and even lost with a ten-fraction regimen [83]. In parallel, treatment planning studies have raised important technical considerations. For example, proton FLASH treatment plans for liver tumours using small fractional doses (∼4.5 Gy) resulted in higher doses to organs at risk compared with conventional stereotactic body radiotherapy (SBRT) plans, underscoring the current challenges of achieving FLASH delivery under conventional fractionation constraints [84]. Taken together, these data indicate that the FLASH effect is most robust under high single-dose or hypofractionated conditions, yet the clinical desirability of such regimens must be balanced against the increased risk of late toxicity, particularly for large treatment volumes [10,85]. As many clinical targets are not amenable to truly ablative single fraction, hypofractionation has emerged as a pivotal strategy to broaden the clinical applicability of FLASH-RT. Further research into the potential dose dependence of the FLASH sparing effect for both normal tissues and tumours under clinical fractionation schemes is needed to better define the limitations and clinical applicability of FLASH-RT.

### 3.4 Radiation modality

FLASH-RT has been investigated across nearly all radiation modalities currently used for clinical radiotherapy, including electrons, photons, protons, and heavy ions. However, the feasibility, reproducibility, and biological manifestation of the FLASH effect are strongly influenced by the physical characteristics of each radiation source, including penetration depth, beam controllability, energy deposition patterns, beam quality and the technical capability to achieve ultra-high dose rates under clinically relevant conditions. Understanding source-specific advantages and limitations is therefore essential for rational clinical translation.

#### 3.4.1 Electrons

Electron beams represent the earliest and most extensively studied radiation source in FLASH-RT research [86]. Electron-based FLASH-RT has been shown to evoke robust and reproducible tissue-sparing effect across various organ systems including the whole brain [28], lungs [37], abdomen [87], skin [10,14,88,89], developing embryos [73] across multiple species such as zebrafish, mice, rats, cat, pig, dog and humans. Despite this consistency, the magnitude of the FLASH effect has varied substantially across studies. To address this issue, Schüler et al. emphasized the necessity of the standardized and detailed reporting of physical parameters including fractionation scheme, total dose, irradiation volume, exposure duration and pulse structure, to enable meaningful cross-study comparisons and reproducibility [86]. A major limitation of electron beams lies in their short penetration depth (typically 2–5 cm), restricting their clinical application to superficial tumours of limited size, or to intraoperative radiation therapy. To overcome this constraint, very high energy electrons (VHEE; ∼100–250 MeV) have emerged as a promising alternative for electron FLASH applications [90,91]. Compared to conventional commonly used X-rays, VHEE beams offer improved depth-dose profiles, enabling irradiation of most deep-seated tumours leveraging their highly penetration nature [2,92]. Early i*n vitro* studies using plasmid DNA damage endpoints have already demonstrated FLASH-RT associated sparing effects with VHEE irradiation [90]. However, due to the lack of clinical applicability evidence and immaturity of technical platforms for VHEE in FLASH research, this approach still has a long way to go.

#### 3.4.2 Photon beams

Megavoltage (MV) photon beams remain the most common radiation modality employed in over 90% of patients due to its deep tissue penetration, sharper penumbra, and robustness to tissue density variations. It is thus likely that MV photons is a promising candidate for FLASH radiotherapy, however, conventional linear accelerators are currently unable to meet to FLASH-RT conditions due to the inherently low efficiency of bremsstrahlung process of electrons to X-rays conversion [93]. To date, few preclinical studies using photon FLASH-RT have been published [93]. Initial investigation of photon FLASH-RT employed synchrotron-generated kilovoltage (kV) continuous (non‐pulsed) beams, which demonstrated that KV photon FLASH could mitigate radiation-induced brain injury in murine whole-brain irradiation models [29]. Nevertheless, such beams have large surface average dose rates which drop steeply from the surface and enable delivery of FLASH dose rates at depths of only up to a few mm, severely constraining clinical applicability [94]. Generating clinically viable MV photon FLASH impose significant challenges, including stringent demands on accelerator power handling, thermomechanical stress, and cooling systems. Notably, researchers from the China Academy of Engineering Physics and Zojo Flash Medical Technology Co., Ltd., have made groundbreaking advances in developing a MV photon FLASH platform. The superconducting linear accelerator can generate 6–8 MV photon FLASH beams at dose rates exceeding 1000 Gy/s. Using this platform, significant normal tissue sparing was demonstrated in preclinical models of thoracic [95], abdomen [48,49], and cranial irradiation, alongside comparable tumour growth delay and improved overall survival [95]. In parallel, TRIUMF developed the first irradiation platform (FIRST) capable of delivering 10 MV X-ray beams using a newly developed UHDR bremsstrahlung source. This system achieved dose rates exceeding 40 Gy/s over field sizes 1 cm down to a depth of 4.1 cm, enabling rigorous dosimetric and biological studies in murine models [96,97]. Given the widespread clinical availability and clinical familiarity of MV photon beam therapy, MV photon-based FLASH-RT represents a particularly attractive pathway toward clinical translation. However, comprehensive biological validation and careful assessment of dosimetric robustness, field size scalability, and normal tissue tolerance remain prerequisites before clinical deployment.

#### 3.4.3 Protons

Compared with conventional photon treatment, proton radiotherapy reduces dose deposition in surrounding normal tissues while precisely targeting the tumour. Excitingly, most clinical proton therapy systems using pencil beam scanning (PBS) can readily achieve or exceed the FLASH dose rate (>40 Gy/s) around individual spot positions for fixed energies, making protons a highly promising modality for FLASH-RT [98]. Preclinical evidence for proton FLASH-RT, however, has been mixed. *In vitro* studies showed no sparing effect at acute endpoints such as cloning survival and γH2AX foci, likely due to experiments conducted under ambient atmospheric conditions (∼21%) rather than physiological tissue oxygenation levels (∼3–5%) [99]. In contrast, in terms of delayed endpoints, proton FLASH has been found to reduce premature senescent and chronic inflammation in normal lung fibroblasts, indicating a long-term benefit [100]. In vivo studies have provided more compelling evidence. While no FLASH effect was observed in zebrafish embryos, significant normal tissue sparing has been reported in rodent models involving the thorax [101], skin [9,102,103], bones [103], abdomen [104], brain [105,106] and heart [58], with tumour control comparable to conventional proton therapy [104,107]. However, negative or adverse findings have also been reported, including the absence of neurocognitive protection following 18 Gy cranial proton FLASH [108], and even increased acute lethality following whole abdomen PBS proton FLASH [51]. These discrepancies underscore the urgent need for full dose-response and dose rate-response curves to define the FLASH factor and the protection ratio of proton FLASH for the same particle type and energy. Furthermore, most proton FLASH studies have focused on the entrance portion of the Bragg peak, whereas clinical proton therapy typically employs spread-out Bragg peaks (SOBP). Initial studies indicated that SOBP FLASH can confer consistent normal tissue sparing effect in tissues such as skin and intestine [17,109]. However, the influence of both the SOBP delivery and the increased Linear Energy Transfer (LET) on the proton sparing effect on normal tissues and tumour control efficacy remains incompletely understood and warrants systematic investigation.

#### 3.4.4 Heavy ions

Heavy-ion radiotherapy, particularly carbon-ion therapy, offers distinct physical and biological advantages due to the elevated relative biological effectiveness (RBE). However, FLASH-RT research using heavy ions remains in its infancy largely due to the scarcity of suitable facilities and technical challenges of achieving FLASH conditions with synchrotron accelerators [2]. Recent technical improvements have begun to address these limitations. The Heidelberg Ion-Beam Therapy Center (HIT) synchrotron achieved dose rates of 185 Gy/s with helium and >40 Gy/s with carbon ions, and demonstrated a significant FLASH sparing effect under hypoxia conditions (0.5%-4% O_2_) *in vitro* [110,111]. In contrast, early human cell studies conducted at the Gunma University Hospital using carbon ions at dose rates up to 195 Gy/s failed to detect FLASH effect under the tested conditions [112]. Despite these inconsistencies *in vitro* models, *in vivo* studies have yielded promising positive results. In zebrafish embryos, helium ions FLASH-RT delivered under elevated oxygen partial pressure (30 mmHg) spared normal body development and reduced spine curvature compared to CONV-RT [113]. In murine models, ultra-high dose rate helium ion irradiation significantly mitigated brain injury, preserving microvascular integrity and suppressing microglial activation while maintaining tumor control [114]. Moreover, carbon ion FLASH-RT has been reported to not only achieve effective tumor control but also significantly reduce lung metastasis compared with CONV-RT [115]. Notably, the high LET of heavy ions is often accompanied by reduced hypoxia dependence, introducing unique mechanistic avenue for exploration distinct from those of low-LET radiation [113]. A chemistry simulation showed that heavy ions FLASH-RT at high LET could result in a generation of early and highly oxygenated conditions within the tumor at the spread-out Bragg peak, potentially enhancing therapeutic ratio by increasing toxicity in tumor [116]. As carbon-ion therapy continues to expand globally, elucidating the radiobiological principles underlying heavy-ion FLASH-RT represents a critical frontier. As highlighted by Weber et al., numerous fundamental questions remain unresolved in this emerging field of carbon ion FLASH [117]. If consistent and reproducible normal tissue sparing be confirmed, it could further improve the therapeutic index of carbon ion therapy and potentially pave the way for the clinical exploration of even heavier ion species.

## 4 Mechanisms of action in FLASH-RT

Despite the rapidly increased experimental evidence supporting the normal tissue sparing effect of FLASH-RT, its underlying biological mechanisms remain incompletely understood. Here, we summarize the main mechanistic frameworks proposed to date, highlighting both the supporting evidence and unresolved controversies. Mechanisms including transient oxygen depletion, free radical chemistry, mitochondrial metabolism, iron-dependent lipid peroxidation, genomic integrity, and modulation of inflammation and immune response have all been shown to contribute to the protection of normal tissue to varying extents. However, none of these mechanisms can independently explain the heterogeneous and even contradictory results observed across different tissues, irradiation modalities, and experimental platforms.

Instead, the existed data might support a conditional, multi-layered model, in which the FLASH effect emerges only within a restricted operational window defined by baseline tissue oxygenation, redox capacity, DNA integrity, mitochondrial metabolic state, vascular architecture, immune response, and precise beam delivery characteristics such as mean dose rate, dose per pulse, and temporal structure mentioned above. This model also provides a rational explanation for the negative or absented FLASH effect studies. The expected sparing effect may be attenuated, abolished, or even reversed when one or more of these permissive conditions are not met.

### 4.1 Radiochemical mechanisms

The FLASH effect is initiated by ultra-fast radiochemical events that serve as the primary drivers of the biological sparing effect. These events operate on a picosecond to microsecond timescale(10^−15^-10^−6^s), establishing the initial biochemical footprint of FLASH-RT. This section examines the two complementary radiochemical hypotheses including the radiolytic oxygen depletion (ROD) and radical recombination, which dictate the magnitude of reactive oxygen species (ROS) production at early stage. We discuss their relative contributions and limitations, emphasizing that while they are the physical prerequisites for FLASH-RT, their impact is also modulated by tissue-specific oxygenation and microenvironments.

#### 4.1.1 Radiolytic oxygen depletion hypothesis

**Fig. 4:**
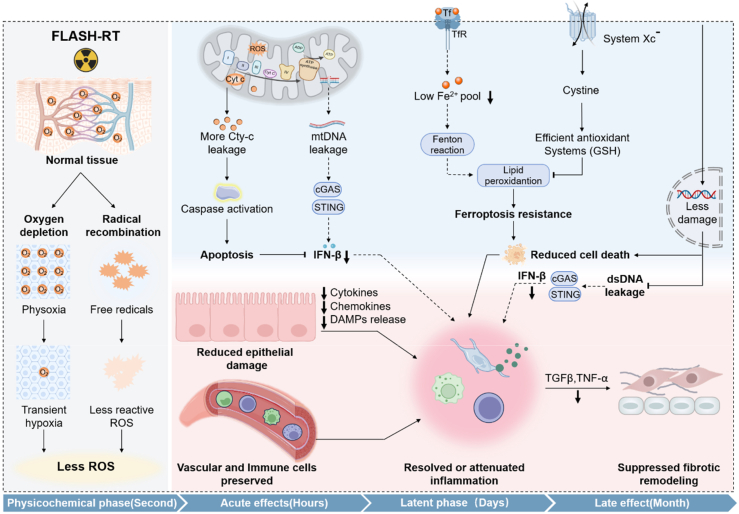
**Mechanistic hypotheses underlying the Normal tissue sparing effect of FLASH-RT.** FLASH effect is initiated by ultra-fast physical events occurring within the microsecond-to-millisecond window (radiolytic oxygen depletion and radical-radical recombination), which serve as the primary triggers. Differential oxygen dynamics during FLASH-RT, where rapid oxygen depletion induces transient hypoxia and high radical recombination in normal tissue, reducing ROS formation. These events (decreases in ROS levels) dictate the subsequent biological response phase, characterized by the attenuation of mitochondrial oxidative stress, inhibition of ferroptosis, and reduced DNA/genomic integrity. Finally, these early molecular events culminate in the systemic output phase (hours to days), where preserved immune cells, vascular niches and minimized inflammatory cascades lead to tissue-level functional recovery. FLASH-RT reduced inflammatory response by preserves immune cells populations, limiting early immune cell depletion and promoting reduced inflammatory cytokine release. In parallel, FLASH-RT attenuates epithelial injury and EMT signaling, maintaining epithelial integrity and restraining fibrotic progression (Dashed lines represent inhibition of the pathway).

Radiolytic oxygen depletion (ROD) has been proposed as a prominent mechanism underlying the FLASH effect [118,119]. During irradiation, free radicals generated by water radiolysis react with molecular oxygen to form oxygen peroxyl radical, thereby consuming oxygen and limiting further oxygen-dependent fixation of DNA damage [120]. The ROD hypothesis posits that the ultra-short radiation pulse of FLASH-RT transiently deplete intracellular oxygen via radiolysis, where reoxygenation cannot occur rapidly enough, leading to a limited accumulation of additional ROS and temporarily increases the radio-resistance in normal tissues [13,121,122] (Fig. 4). Conversely, due to the pre-existing hypoxic microenvironment in tumor, the reduction in oxygenation induced by FLASH-RT is insufficient to further alter tumor radiosensitivity (Fig. 4) [123]. This differential biological lethality between normoxic and hypoxic conditions is caused by oxygen enhancement ratio (OER) [124]. Decades ago, Dewey and Boag first established that short and high-intensity electron pulses reduce radiosensitivity by inducing transient hypoxia via rapid oxygen consumption [125]. Subsequent studies has explored the FLASH effect across various oxygen levels in multiple biological systems, including bacteria [125,126], mammalian cell lines [[127], [128], [129]], and small animals such as mice and zebrafish [28,130]. Most *in vitro* studies reported a significantly augmented cell survival under hypoxic conditions (e.g., 1.6%, 2.7%, 4.4% O_2_) following FLASH-RT, whereas no significant difference was observed under normoxia [28,111,129,131]. Cooper et al. showed that DNA damage reduction by FLASH-RT is oxygen-dependent, as observed in whole blood samples under severe hypoxia (0.25–0.5% O_2_) [70]. The FLASH effect has been consistently observed in zebrafish embryos even under hypoxia [69,132], yet it is attenuated by hyperoxygenation. For example, increasing brain oxygenation via carbogen breathing or oxygen supplementation during anesthesia diminishes the FLASH sparing effect, highlighting its oxygen-dependent nature [28,106]. Chemical and biophysical evidence further supports the ROD hypothesis. Kusumoto et al. reported dose rate-dependent reductions in the production of 7-OH-C3CA, which can be generated through an oxygen-consuming reaction during radiation, implying rapid oxygen consumption under FLASH-RT [133]. Consistently, an intracellular oxygen transient quantification *in vivo* via protoporphyrin IX (PpIX) delayed fluorescence also depicted an immediate reduction in oxygen levels corresponds to FLASH beam delivery, the extent of which is seen to increase with the dose and depends on the initial oxygen levels [134].

However, the oxygen depletion hypothesis also has been challenged. Several *in vitro* studies reported normal tissue protection by FLASH-RT even under normoxic conditions [135], and *in silico* models have shown that oxygen in most normal tissues is unlikely to be depleted to radioprotective level by FLASH dose rate [[136], [137], [138]]. Direct measurements techniques such as solid optical sensors and phosphorescence or fluorescent quenching are commonly employed in experimental verification, which demonstrated that oxygen was not consumed to radiologically relevant hypoxia levels during FLASH-RT [75,121,[139], [140], [141]]. Intriguingly, Cao et al. reported even lower oxygen consumption during FLASH compared with CONV-RT [121]. Another study showed that oxygen levels can be depleted by amounts that are sufficient to affect radiosensitivity only at intermediate initial oxygen tension (termed “physoxia”, a condition relevant to many normal tissues), while no effect was elicited at high or very low initial oxygen levels [142,143]. This is consistent with reports that FLASH effect was reversed in the absence of sufficient oxygen in mouse skin, supporting the fact that FLASH sparing emerges only within a restricted oxygen window, which requires intermediate, rather than extreme tissue pO_2_, emphasizing the importance of tissue-specific baseline pO_2_ in the FLASH-induced protection [144,145].

In summary, although radiolytic oxygen depletion oxygen as an important determinant of FLASH-induced tissue protection, it is unlikely to be the standalone mechanism. Tissue specific physiological parameters including baseline pO_2_, intercapillary spacing, vasculature geometry/density may play critical roles in determining FLASH effect [143,146,147]. Developing accurate and interpretable measurements of oxygen transients *in vivo* are crucial for validating mechanistic models. Notably, the transient oxygen depletion might also simultaneously confer increased radio-resistance to tumour tissues. Although some studies suggested that FLASH provided comparable tumour control in both acutely hypoxic and normoxic tumours, comprehensive evaluations combining normal tissue protection and tumour suppression under varied oxygen tensions are still lacking and more preclinical research is needed to address these gaps [106].

#### 4.1.2 Radical recombination hypothesis

**Fig. 5:**
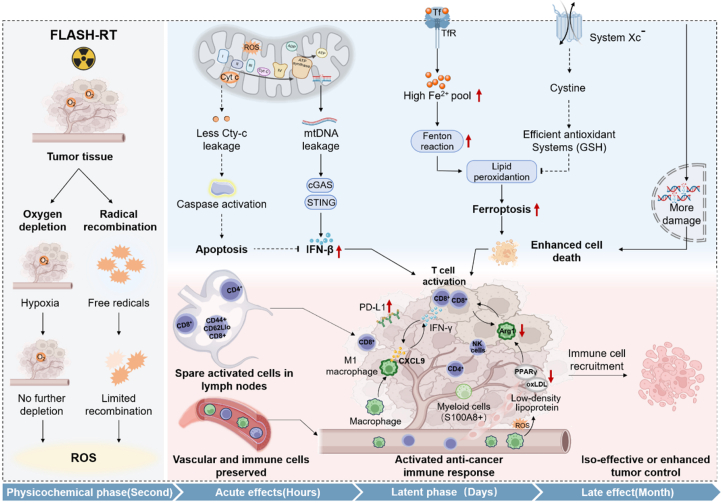
**Mechanistic hypotheses underlying the tumor control of FLASH-RT.** Due to the pre-existing hypoxic microenvironment in tumor, the reduction in oxygenation induced by FLASH-RT is insufficient to further alter the radiosensitivity. The limited recombination further inhibited ROS reduction. These events (Increased in ROS levels) activated the subsequent biological response phase, characterized by the increased mitochondrial oxidative stress, ferroptosis, and more DNA damage. These injuries further activate the downstream anti-tumor immune response. FLASH-RT preserves tumor vasculature, maintaining vessel morphology and CD31 expression. The preserved vasculature further facilitates immune cell infiltration and enhances lymphatic drainage. FLASH-RT activates T cells, including CD8^+^ and CD4^+^ T cells, and induces a shift in macrophage polarization from the pro-tumor M2-like phenotype to the anti-tumor M1-like phenotype. This immune activation leads to enhanced tumor control, with a reduced presence of regulatory T cells (Tregs) and lower PD-1 expression, reflecting a favorable anti-tumor immune environment. The diagram highlights the complex interactions between immune cells, tumor cells, and the vasculature, illustrating how FLASH-RT modulates the TIME to optimize anti-tumor immunity while sparing normal tissues (Dashed lines represent inhibition of the pathway).

An alternative and complementary explanation for the FLASH effect is the altered free radical chemistry [148]. Indirect radiation damage is primarily mediated by water radiolysis, producing free radicals such as hydrogen (H•) and hydroxyl (OH•) radicals. These species can then react with biological molecules (RH) and oxygen to yield organic peroxyl radicals ROO•, which drive lipid peroxidation and DNA damage, and ultimately cell death. Importantly, the radical species (R• and ROO•) can also undergo self-recombination reactions that competing with the damaging peroxidative chain reactions. Labarbe et al. proposed the free radical recombination hypothesis for FLASH-RT by mathematical simulation of the free radical reaction equation. In this framework, FLASH-RT is believed to generate extremely high transient concentration of radicals within an ultra-short time window, leading to an elevated proportion of radical recombination and less damaging peroxyl radicals (Fig. 4, Fig. 5), [138]. However, this hypothesis ignored the complex chemical environment within the cytoplasm and failed to explain the differential effects between normal and tumour tissues. Hu et al. further expanded the radical recombination model by incorporating endogenous antioxidant capacity [149]. They proposed that the higher endogenous antioxidant levels in tumor facilitate the preferential scavenging of peroxyl radicals, thereby suppressing radical recombination and rendering tumour cells more vulnerable to FLASH-RT compared to normal tissues (Fig. 4, Fig. 5) [149]. Although direct experimental verification remains challenging due to the ultrafast timescale of radical reactions (10^−12^–10^−6^ s), indirect evidence supports this hypothesis. Experiments in simplified aqueous systems have demonstrated a dose-rate-dependent decrease in the G value (radiation chemical yields) of free radicals and H_2_O_2_ [77,150]. Furthermore, lipid peroxidation is a critical free radical reaction that disrupts physiological homeostasis. Portier et al. showed that downregulation of oxylipins, formed by the free radical reaction of polyunsaturated fatty acids, was a hallmark of FLASH irradiation in normal cells [151]. While these intermediate product measurements provide crucial empirical support, definitive validation requires the development of high-precision, instantaneous radical probes capable of operating within the complex intracellular matrix, rather than simplified water systems.

In summary, ROD and radical recombination are parallel, competing processes in radiation chemistry. Under the ultra-high dose rate pulses of FLASH-RT, ROD creates a transiently oxygen-depleted microenvironment. Simultaneously, the resulting high radical density further facilitates radical-radical recombination, thereby suppressing the formation of damaging peroxyl species. These two mechanisms may act synergistically and together serve as the upstream prerequisite for the downstream biological protective effects. Regarding their relative contribution to the FLASH effect, the study by Rothwell et al. compared DNA damage reduction *in vitro* with oxygen depletion for FLASH-RT modelled *in silico*, confirming that ROD plays a significant role in reducing DNA damage under low oxygen conditions. Notably, however, both the simulation parameters and experimental data suggest that ROD may only partially account for the broad FLASH-sparing effects observed *in vivo* [152]. Consequently, further investigations that simultaneously address oxygen dynamics and radical kinetics within physiologically relevant cellular milieus are essential. Such studies will be critical for elucidating the relative and synergistic contributions of these processes and for guiding the rational optimization of FLASH radiotherapy.

### 4.2 Radiobiology mechanisms at the cellular level

Following the initial radiochemical perturbation, the sparing signal is propagated and amplified through the stabilization of genomic integrity and intracellular organelles. This section integrates DNA damage signaling, mitochondrial metabolism, and iron homeostasis as secondary mediators that translate the initial physical attack into cellular response. We propose that these pathways are not independent. They form a functional network wherein the preservation of mitochondrial and genomic stability collectively prevents the activation of apoptotic and pro-inflammatory cascades, thereby becoming the core for maintaining the FLASH effect at the cellular level.

#### 4.2.1 DNA integrity and damage signalling

Radiation-induced DNA damage is primarily caused by the direct ionization of the DNA backbone and the indirect attack of reactive free radicals generated from the radiolysis of water, a process further amplified by oxygen acting as a damage-fixation agent. Radiation-induced chromosome breaks also lead to the leakage and accumulation of DNA fragments into the cytoplasm, activating the cGAS-STING pathway and type I interferon (IFN-I) release. This signalling leads to the recruitment of CD8^+^ T cells and initiates immune and inflammatory responses. Shi et al. found that FLASH-RT elicited notably less cytoplasmic dsDNA and attenuated cGAS-STING signalling in the intestinal crypts than CONV X-ray, resulting in a diminished cascade of CD8^+^ T cell chemotaxis and gasdermin E-mediated pyroptosis. In contrast, FLASH X-ray was as competent as CONV-RT in boosting the antitumor response evoked by dsDNA signalling [48]. The “DNA integrity” hypothesis was proposed to explain this differential effect. Specifically, CONV-RT caused sustained DNA damage and compromised genomic integrity, leading to the accumulation of fragmented DNA and activated cGAS pathway. In contrast, ultra-fast dose deposition of FLASH-RT reduced the occurrence of DNA breaks and generation of DNA fragments, resulting in lower cGAS-STING signaling in normal tissues [48](Fig. 4). Consistently, multiple studies report reduced γH2AX and 53BP1 foci, diminished senescence, and lower inflammatory gene expression following FLASH-RT in normal cells [38,100]. FLASH-RT also induced less DNA damage than CONV-RT in peripheral blood lymphocytes (PBLs), a effect modulated by oxygen tension, total dose, and dose rate [70]. Studies using plasmid DNA also confirmed that FLASH-RT produced reduced amounts of DNA single-strand breaks (SSBs) [90]. Conversely, DNA damage in tumor tissues appears primarily dose-dependent, largely independent of dose rate. To fully elucidate the FLASH effect from the perspective of genomic integrity, it is imperative to delineate the specific patterns of DNA lesions and the corresponding repair pathways provoked by FLASH versus CONV in both normal and tumor cells, and to further clarify how these distinct damage–repair dynamics shape the subsequent antitumor immune response. In summary, FLASH-induced mitigation of DNA damage, stemming from the suppressed production of early-stage free radicals, does more than boost cell survival. Importantly, it preserves cellular signaling integrity, safeguarding the activation of effective immune responses.

#### 4.2.2 Mitochondrial metabolism

As the primary organelles consuming oxygen and producing ROS within cells, mitochondria play a central role in directing radiation-induced signal transduction, cell death, and immune responses [153]. This effect is dependent on both radiation dose and quality [[154], [155], [156], [157]]. Ionizing radiations induces excessive oxidative stress and disrupts mitochondrial homeostasis, resulting in the cytosolic leakage of cytochrome *c* (cyt *c*) and mitochondrial DNA (mtDNA). These events subsequently activate caspase-dependent apoptosis and the cGAS-STING mediated type-I interferon response. Recently, studies suggested that the antagonism between cytosolic cyt *c* and mtDNA in cell fate and immune response is crucial to the therapeutic efficacy of FLASH-RT. Han et al. demonstrated that cyt c−/− mouse embryonic fibroblasts exhibit reduced apoptosis and necrosis following FLASH-RT compared with wild-type counterparts [158]. To further investigate the mitochondrial basis of the FLASH effect, Lv et al. conducted inspiring study to unveil the regulation of mitochondria-mediated apoptosis and inflammatory signaling by FLASH-RT. They demonstrated that in non-tumorigenic MCF-10A cells, FLASH-RT supressed cytosolic mtDNA accumulation and subsequent IFN-β secretion by enhancing cyt *c* release and caspase activation [123,159,160]. In contrast, carcinoma cells MDA-MB-231 exhibited limited cyt *c* release, leading to increased cytosolic mtDNA accumulation and IFN-β secretion [160]. Electron transport chain (ETC) disruption hypothesis was proposed to explain this differential response. The model suggested that FLASH-RT stimulated the cascade feedback from ETC dysfunction to cyt *c* leakage, enhancing intrinsic apoptosis while suppressing inflammation signalling in normal tissues (Fig. 4). Simultaneously, because many tumour cells rely on aerobic glycolysis (Warburg effect) and exhibit diminished ETC activity, FLASH-RT can still disrupt the ETC function of tumour cells but failed to stimulate the positive feedback of cyt *c* leakage (Fig. 5) [160]. Moreover, FLASH-RT has also been shown to preserve mitochondrial morphology in normal cells, an effect associated with reduced dephosphorylation of the Dynamin-1-like protein [161]. In contrast, in carcinoma cells, FLASH-RT enhanced mitochondrial fission and reduced morphology network complexity, while no such effects were observed in normal breast cells [160]. Taken together, mitochondrial function has emerged as a critical determinant of the FLASH effect. However, it remains unclear whether the mitochondrial perturbations induced by FLASH-RT are merely transient events or whether they trigger long-term mitochondrial remodeling that contributes to sustained protective effects. Moreover, the interplay between mitochondrial apoptosis and autophagy, particularly mitophagy, and inflammation under FLASH conditions remains largely unexplored.

#### 4.2.3 Iron metabolism and ferroptosis

Iron is vital in cellular respiration, DNA synthesis and various metabolic functions. Iron metabolism has recently emerged as another critical determinant of FLASH-RT induced differential responses between normal and tumour tissues. During irradiation, superoxide reacts with iron-containing proteins (i.e., aconitase, ferritin, Fe-S proteins) to release redox-active Fe^2+^, amplifying oxidative damage through the generation of additional HO• and RO• through Fenton reactions [162]. This free radical chain reactions can triple the size of the intracellular redox-labile iron pools during irradiation. Tumour cells, which typically exhibit elevated levels of Fe^2+^ and transferrin receptors, are inherently more vulnerable to this radical burst induced by irradiation [163,164]. However, normal cells are protected by lower iron availability and more effective antioxidant system that sequester Fe^2+^, thereby curtailing peroxidation reactions and limiting collateral damage [162]. Nuria et al. provided the first *in vivo* evidence linking iron metabolism to the FLASH effect, demonstrating that FLASH-RT selectively increased lipid peroxidation and ferroptosis in tumor cells, with no significant increase observed in normal tissues compared with CONV-RT(Fig. 4, Fig. 5) [165]. Importantly, raising iron levels in normal tissues through high-iron diet abolished the FLASH sparing effects, highlighting that the baseline iron status are critical determinants in mediating the protective outcomes of FLASH-RT [162,165]. Despite this evidence, *in vivo* studies investigating the direct impact of FLASH-RT on iron metabolism remain scarce. Further research is needed to delve into FLASH effect in different iron-containing tissues to elucidate the relationship between ferroptosis and lipid metabolism under FLASH dose rates. It is also essential to verify whether high levels of labile Fe^2+^ and transferrin in tumours contribute to the targeted elimination of tumours by FLASH-RT while selectively protecting normal tissues with opposite iron-related characteristics.

### 4.3 Tissue-level responses: vascular protection and stem cell preservation

The translation of cellular protection into tissue-level sparing relies on the maintenance of structural and regenerative integrity. At the tissue level, the normal tissue sparing effects of FLASH-RT are increasingly attributed to the preservation of vascular integrity and regenerative cell populations, both of which are essential for maintaining tissue homeostasis and functional recovery after irradiation. Transcriptomic analysis of irradiated skin demonstrated that FLASH-RT preferentially promote the upregulation of tissue and vascular repair pathways [103]. Persistent vasculature abnormalities also contribute to an altered CNS microenvironment that further compromises the integrity of the blood-brain barrier. Allen et al. showed that compared to isodoses of CONV-RT known to elicit detrimental effects, FLASH does not damage the normal vasculature, providing the first evidence that FLASH preserves microvasculature integrity in the brain [166]. Consistently, FLASH has also been shown to maintain the microvasculature of the BBB in radiosensitive juvenile mice models, potentially through the preservation of aquaporin 4(AQP4) [31].

In parallel, accumulating evidence indicates that FLASH-RT preserves tissue-resident stem and progenitor cell compartments, which are central to post-irradiation regeneration. FLASH-irradiated skin patches retained higher populations of CD34 and Lgr6^+^ epidermal stem cells [10,103]. Similarly, FLASH dose rate significantly preserved the proliferation kinetics in crypt cell regeneration compared to CONV-RT by sparing the Lgr5^+^ crypt base columnar (CBC) cells [47]. In addition, the higher fractions of radiosensitive stem and progenitor cells in within neurogenic niche is also the key attributors of radiation induced brain damage and cognitive impairment. FLASH-RT spared the neurogenic niche and hippocampal neurogenesis supported by the retention of both newly born immature (DCX^+^) and mature (NeuN^+^) neuronal populations in the hippocampal dentate gyrus after FLASH-RT [32]. However, it also raises a critical and underexplored concern that whether FLASH-RT may preserve cancer stem cells (CSCs). CSCs are widely recognized as key drivers of radio-resistance, tumor recurrence and therapeutic failure. Emerging evidence suggests that CSCs are more resistant than normal cancer cell to FLASH-RT, which may associate with the increase of lysosome-mediated autophagy, and the decrease of apoptosis, necrosis and pyroptosis [167]. This suggests that FLASH effect may be governed not strictly by tissue type, but also by cellular state. Specifically, FLASH-RT may preferentially spare cells with high antioxidant capacity, metabolic flexibility and stress resilience, the features shared by both normal stem/progenitor cells and CSCs. Consequently, future studies should focus on CSC-specific endpoints rather than relying solely on bulk tumor response. Moreover, rational combination strategies, such as the integration of FLASH-RT with autophagy inhibitors or CSC-targeting therapies, may be required to mitigate potential tumor-protective effects while preserving normal tissue sparing.

### 4.4 Systemic responses: inflammation and immune modulation

The ultimate manifestation of FLASH-RT lies in its systemic responses, characterized by dampened harmful inflammation and remodeling of antitumor immunity. This section discussed how FLASH-RT minimize early-stage tissue damage, preventing the amplification of the systemic inflammatory response, while simultaneously reshaped the tumor immune microenvironment (TIME) to favor antitumor efficacy, thereby achieving therapeutic benefit. Crucially, the immune regulation is not isolated event but an integrated response resulting from preserved signaling homeostasis and the suppression of inflammatory triggers that would drive immune suppression and tissue fibrosis.

#### 4.4.1 Attenuation of radiation-induced inflammation in normal tissues

Ionizing radiation frequently provokes excessive ROS generation, DNA damage and cell death, collectively triggering prolonged and dysregulated inflammatory cascades. Such maladaptive inflammation represents a primary driver of acute and late toxicities in normal tissues, ultimately compromising organ function and patient quality of life. A growing body of preclinical investigations have demonstrated that FLASH-RT could significantly attenuates inflammation, fibrosis, and organ dysfunction across multiple normal tissues [168]. Compared with CONV-RT, FLASH irradiated mice exhibited reduced depletion of white blood cells and lymphocyte, suggesting enhanced preservation of immune homeostasis [49]. In addition, the reduced recruitment of innate immune cells including neutrophils and macrophages has been consistently observed in intestine and skin following FLASH-RT [103,169]. This protective effect was further supported by cytokine profiling, which showed significantly lower levels of pro-inflammatory cytokines (TNF-α, IL-6, and IL-10 et al.) especially in late-stage post irradiation, indicating a diminished and sustained detrimental inflammatory signalling after FLASH-RT [37,49,100,103](Fig. 4). In the central nervous system, FLASH-RT also has been shown to markedly reduce astrogliosis and microglial activation compared with CONV-RT, indicating attenuated neuroinflammation [28,29,81]. Although both triggered the complement cascade, downstream signalling diverges. FLASH-RT failed to induce expression of endogenous damage-associated molecular pattern (DAMP) receptors on astrocyte surface, thereby limiting sustained inflammatory amplification in irradiated brain tissue [81].

Radiation induced epithelial–mesenchymal transition (EMT) is another critical contributor to chronic fibrosis and organ dysfunction following CONV-RT. Endothelial and epithelial damage from CONV-RT release DAMPs and soluble immune mediators, activating inflammatory and pro-fibrotic pathways, resulting chronic fibrotic changes and organ dysfunction linked to radiation-induced EMT. However, recent advances indicated that FLASH-RT could suppress EMT-associated pathways, thereby reducing toxicity and fibrosis to normal tissues by maintaining epithelial properties and modulating EMT regulators such as TGF-β and mesenchymal markers including vimentin and N-cadherin (Fig. 4) [170]. Intriguingly, FLASH-RT induced modulation of EMT appears to be temporally dynamic. FLASH irradiation enhanced TGF-β signal and EMT in alveolar type 1 cells during the acute phase (up to 7 days post irradiation), coinciding with accelerated inflammation resolution and tissue repair [44]. Whether this early activation is self-limiting or persists to drive long-term fibrosis remains unresolved. Future studies with extended follow-up and comprehensive fibrotic endpoints are needed to define the durability and functional consequences of these acute responses. These findings suggested that the normal tissue-sparing effect of FLASH-RT is closely linked to attenuated inflammation amplification and preserved immune system viability, although the precise molecular hierarchies governing these effects remain incompletely defined.

#### 4.4.2 Tumour immune microenvironment modulation

Radiotherapy reshapes the tumor immune microenvironment (TIME) by modulating the balance between immune-stimulatory and immune-suppressive signals. This process is influenced by irradiation parameters, including beam quality, fractionation, and delivery configurations such as dose rate and spatial distribution. Advanced modalities such as proton/heavy ion radiotherapy [171,172], stereotactic body radiation therapy (SBRT) [173], spatially fractionated radiation therapy (SFRT) [174], and low-dose radiotherapy(LDRT) [175] have been shown to effectively reprogram TIME to reverse immune desertification and overcome immunotherapy resistance. Similarly, FLASH-RT has emerged as another potential immunomodulatory approach capable of preserving normal tissue while reshaping TIME [2], suggesting that immune activation is a critical driver of its antitumor efficacy.

##### 4.4.2.1 Immune cell reprogramming and cytokine remodeling

Radiotherapy is a dual-edged sword in modulating tumour immunogenicity. While it can stimulate anti-tumour immunity by triggering the release of inflammatory mediators, increasing the infiltration of immunostimulatory cells, it can also suppress anti-tumour immunity by promoting the recruitment of regulatory T cells (Tregs) and myeloid-derived suppressor cells (MDSCs) [176]. The balance between pro- and anti-tumour outcomes is partially regulated by the profile of radiotherapy-induced chemokine and cytokine profiles [177]. Emerging evidence from various tumour models suggested that FLASH-RT might promote a more antitumor immune microenvironment. Studies in ovarian and lung cancer have demonstrated enhanced infiltration of endogenous T cells (CD8^+^, CD4^+^), myeloid cells (S100A8^+^), and polarization of macrophage from pro-tumorigenic M2-like phenotypes toward antitumor M1-like phenotype following FLASH-RT (Fig. 5) [87,178,179]. These changes were concomitant with a greater reduction in Tregs and PD-L1 expression, indicating a more robust antitumor immune response and contributing to superior tumour control relative to CONV-RT [179]. Mechanistic insights further indicated that FLASH-RT may reprogram macrophage lipid metabolism to reverse tumour immunosuppression, as shown in medulloblastoma models treated with proton FLASH [180]. Intriguingly, FLASH-RT was reported to overcome radioresistant head and neck squamous cell carcinoma (HNSCC) by activating CD8^+^ T cells, which in turn stimulated M1 macrophages through paracrine IFN-γ signal. The activated M1 macrophages secreted CXCL9 to re-activated CD8^+^ T cells, establishing a feedforward loop that amplified TIME activation. However, this phenomenon was not observed in radiosensitive tumours (Fig. 5) [181]. FLASH-RT also has been shown to spare activated T cells in tumour-draining lymph nodes while increasing checkpoint inhibitor expression within tumours, highlighting its potential synergy with immune checkpoint blockade [182]. Long-term immunological memory of FLASH-RT also has been evidenced by the rejection of tumour rechallenge in cured animals [183]. Radiation-induced lymphopenia also is a well-established negative prognostic factor for patients undergoing radiotherapy [184,185]. It has been hypothesized that the FLASH effect may, in part, be attributable to reduced radiation exposure and preservation of circulating blood and lymphocytes, which has been validated in tumor-draining lymph nodes [182,186,187](Fig. 5).

However, the immunological outcomes of FLASH-RT are not universally superior. Comparable immune infiltration and antitumor efficacy between FLASH-RT and CONV-RT have been reported in diffuse midline glioma [32], situ breast cancer [169], and systematic multi-model evaluations across varying immune competencies mice [188]. In orthotopic glioma rat models, conventional proton irradiation induced stronger immune cell infiltration than FLASH-RT [107]. These inconsistent results suggest that the immunological benefits of FLASH-RT are not universal and likely exhibit model-dependent variability, with factors such as tumour types, the baseline of tumour immunogenicity, implantation site, linear energy transfer (LET), single dose, dose rate, and their complex interactions may all potentially influencing the characteristics of TIME elicited by FLASH-RT (Table 3).

**Table 3:** Researches on tumor immune microenvironment modulated by FLASH-RT.

Model	Radiation parameters	Immune markers assessed	Primary outcomes	Statistical significance	Ref
Subcutaneous Lewis Lung Carcinoma	Proton (15 Gy)	S100A8^+^, CD8α	Increased myeloid cell and cytotoxic T cell infiltration in tumours	Significant (p < 0.05)	(178)
	FLASH: 352 ± 4 Gy/s;				
	CONV: 0.06 ± 0.001 Gy/				
Orthotopic Lewis Lung Carcinoma	Proton (18 Gy)	CD45^+^,CD3^+^,FOXP3^+^,CD8^+^,CD4^+^, F4/80^+^, CD163^+^, PD-1/PD-L1	Increased M1-like macrophages,CD8^+^ T cell infiltration, reduced T-regs, M2-like macrophages and PD-1/PD-L1 pathway activation	Significant (p < 0.05)	(179)
	FLASH: 60 Gy/s;				
	CONV: 1 Gy/s				
Ovarian cancer mouse model	Electrons (14 Gy);	CD45, CD4, CD8,Tregs, B cells, macrophages, MDSCs, M1/M2 macrophage, Neutrophils, Ki-67^+^ T cells	FLASH-RT increased intratumoral T-cell infiltration at early timepoints compared to CONV	Significant (p < 0.05)	(87)
	FLASH: 210 Gy/s				
	CONV: 0.126 Gy/s				
Genetically engineered medulloblastoma	Proton (18 Gy)	CD45^+^,CD86^+^,CD206^+^, CD3^+^, CD4^+^, CD8^+^, F4/80^+^, IL-1β, TNF	Increased M1-like Mφ and decreased M1-like Mφ population. enhanced the infiltration of CD3^+^ T cells, and the ration of CD8^+/^CD4^+^	Significant (p < 0.05)	(180)
	FLASH: 97-143 Gy/s;				
	CONV: 0.7-0.9 Gy/s				
Subcutaneous RT-resistant HNSCC	X-ray (6/4Gy);	CD8A^+^,F4/80^+^, CD11b,CD11c, CD206, CD3^+^, CD4^+^, CD8^+,^ CD86^+,^ NK1.1^+,^ CD45^+^, IFN-γ, CXCL9	Increased the proportions of CD8^+^ T cells and M1-type macrophages and decreased the M2-type macrophages	Significant (p < 0.05)	(181)
	FLASH: 88 Gy/s;				
	CONV: 3.095 Gy/min				
Subcutaneous glioblastoma(re-challenge experiment)	Electron (8 Gy×2)	GM-CSF, ICAM-1, IL-2, IL-6, IL-18, TIMP-1, TNF-alpha and VEGF in serium	Cured animals could reject tumor re-challenge	Significant (p < 0.05)	(183)
	FLASH: 88 Gy/s;				
	CONV: 8 Gy/min				
Orthotopic murine model of brainstem DMG	Electrons (15 Gy);	7AAD,CD11B,CD163,CD206,CD11C, MHC-II, CD3, CD8, CD4, GR1, CD19, and IFN1 a/b, CD45	FLASH and CONV induce differential MAC and DC IFN1	Significance (p < 0.05)	(32)
	FLASH: 90 Gy/s;				
	CONV: 2 Gy/min				
Orthotopic Glioma Rat ModelT	Proton (25 Gy);	CD4^+,^ CD8^+^, Tregs, NK cells, B cells, CD8^+^ macrophages, cDC1, Neutrophils	CPT dose rate managed to increase immune cell density in the tumor microenvironment more efficiently, while FLASH dose rate only increased the density of TILs.	Significant (p < 0.05)	(107)
	FLASH: 257 Gy/s;				
	CONV: 4 Gy/s				
Subcutaneous and orthotopic tumor of lung adenocarcinoma and glioblastoma	Electrons (20Gy, 3×8 or 2 × 6 Gy);	CD4^+^, CD8^+^,Tregs, NK cells, B cells, DCs, Macrophages, Monocytes, Granulocytes	FLASH or Conventional Dose Rate Irradiation Involves Equivalent Immune Responses T	No significance	(188)
	FLASH: >2000 Gy/s;				
	CONV: 0.1 Gy/s				
Subcutaneously with Py8119 and Py230 breast tumor	Electrons (10 Gy)	CD4^+^, CD8^+^,CD3^+^,	Similar tumor control efficiency and tumor immune response were observed in the FLASH-RT and CONV-RT groups.	No significance	(161)
	FLASH: 125 Gy/s;				
	CONV: 0.2 Gy/s				

##### 4.4.2.2 Tumor angiogenesis and vascular integrity

Tumor tissues secrete high levels of pro-angiogenic factors, leading to formation of structurally abnormal vasculature that impedes the immune infiltration and therapeutic efficacy [[189], [190], [191]]. Conventional photon radiotherapy has been shown to stimulate the secretion of pro-angiogenic factors such as VEGF and promote tumour angiogenesis and metastasis in a dose-dependent manner [[192], [193], [194], [195], [196], [197]], whereas high-LET modalities such as carbon ion radiotherapy may offer an advantage by reducing tumour angiogenesis and metastasis [198]. Currently, the advantages of FLASH-RT in tumour angiogenesis have been investigated. Kim et al. reported that a single high dose of 15 Gy CONV-RT induced rapid and reversible vascular collapse in transplantable tumours. In contrast, FLASH-RT preserved vessel morphology and CD31 positive area comparable to non-irradiated control group [178]. Mechanistically, compared to FLASH-RT, CONV-RT leads to significantly increased activation of myosin light chain (p-MLC) in tumour cells, accompanied by elevated γH2AX formation. Inhibition of MLC activation abrogated radiation induced p-MLC and γH2AX dynamics, resulting in enhanced immune cell infiltration and tumour microenvironmental changes similar to those observed with FLASH-RT (Fig. 5) [178]. Given the fact that tumour vessel collapse caused by solid stress (compressive and tensile) may promote immune suppression in tumour microenvironment through MDSCs recruitment and PD-L1 upregulation, vascular preservation by FLASH-RT may underpin some of the distinct immune microenvironmental changes [[199], [200], [201]]. Currently, research on FLASH-RT-induced changes in tumour angiogenesis and its subsequent effects on metastasis remains limited.

##### 4.4.2.3 Effect on metastatic colonization of tumor

Notably, except local tumor control, the deleterious effect of radiotherapy that inadvertently promote metastatic outgrowth, has been demonstrated both in human patients and pre-clinical mouse tumour models [202]. Consistently, FLASH-RT is not exempt from this risk. A recent study showed that FLASH-RT induced acute lung injury could promote metastatic colonization. They suggested that the neutrophils recruitment and neutrophil extracellular trap (NET) formation after acute inflammatory response facilitated to metastatic colonization and cancer progression. Pharmacological inhibition of neutrophils with anti-Ly6G antibody markedly reduced metastatic tumour burden following both FLASH and CONV irradiation [45] This result highlighted that despite the robust normal tissue-sparing effect of FLASH-RT, the influence on tumour invasion and metastasis remains incompletely understood and warrants careful evaluation in future longitudinal and prospective studies.

In all, a pivotal question remains whether the immunomodulatory effects of FLASH-RT represent an independent, parallel mechanism or the integrated output of upstream radiochemical and molecular events. We postulate that immunomodulation is best viewed as the biological culmination of a hierarchical cascade. Specifically, the ultra-fast physical-chemical processes, including ROD and enhanced radical recombination, act as the upstream drivers. These events reduce primary DNA damage and mitochondrial stress, thereby restraining the aberrant activation of stress-response pathways (e.g., cGAS-STING) and limiting the pathological amplification of inflammation. Consequently, the distinct antitumor immune microenvironment (TIME) observed after FLASH-RT is likely not an isolated phenomenon, but rather the downstream consequence of preserved cellular integrity and attenuated inflammatory signaling. This integrated perspective suggests that FLASH-RT creates a more favorable immunological state might by minimizing the immunodestructive collateral damage seen in CONV-RT, rather than by triggering a distinct immunomodulatory pathway.

### 4.5 A spatiotemporal hierarchy of the FLASH effect

To synthesize these disparate hypotheses into a unified framework, we propose a spatiotemporal hierarchy of the FLASH effect. In this model, the FLASH effect is initiated by ultra-fast physical events occurring within the microsecond-to-millisecond window (radiolytic oxygen depletion and radical-radical recombination), which serve as the primary triggers. These events (decreases in ROS levels) dictate the subsequent biological response phase (seconds to minutes), characterized by the attenuation of mitochondrial oxidative stress, inhibition of ferroptosis, and preservation of DNA/genomic integrity. Finally, these early molecular events culminate in the systemic output phase (hours to days), where preserved stem cell/vascular niches and minimized inflammatory cascades lead to tissue-level functional recovery. In this hierarchy, reduced DNA damage acts as a direct consequence of initial radical scavenging, while immunomodulation emerges as an integrated output resulting from the preservation of homeostatic signaling and the avoidance of chronic, maladaptive inflammation (Fig. 4, Fig. 5). This spatiotemporal progression explains why parameter variations, such as dose-per-pulse or temporal structure, can fundamentally alter the outcome by disrupting the synchronization of these cascade stages. However, the current mechanism hypotheses are unable to provide a strong explanation for the differences in responses between tumours and normal tissues. The systemic protective effects have not been observed in tumor tissues, indicating that mechanism research should focus more on the differences in the microenvironment and biological matrix between normal tissues and tumor tissues.

In summary, these findings underscore the need to move from phenomenological descriptions to quantitative and mechanistically FLASH study. Inherently, the FLASH effect is a systemic, synergistic response of biological entities to the extreme physical stimulus of ultra-high dose rates. Therefore, mechanistic inquiries should not be confined to a single dimension; rather, they must construct a comprehensive process that spans from physicochemical interactions to macroscopic biological responses. Future research should strive to break down disciplinary silos through multi-modal cross-validation, aiming to dissect the underlying logic linking free radical kinetics, microenvironmental remodeling, genomic homeostasis, and immune regulation. Such an integrated approach is essential for establishing a robust theoretical foundation to guide the precise clinical translation of FLASH-RT.

## 5 Clinical translation and challenges of FLASH-RT

### 5.1 Clinical implementation of FLASH-RT

The progressive improvements of FLASH-RT techniques, coupled with compelling preclinical evidence, have supported the clinical translation of FLASH radiotherapy worldwide. These efforts aim to evaluate the feasibility, safety, and preliminary efficacy of this ultra-high dose rate irradiation paradigm in human patients (Table 4). The first clinical trial of FLASH-RT was reported in 2019 at Lausanne University Hospital. Using a 5.6-MeV linac (Oriatron eRT6), a single 15 Gy fraction of electron FLASH irradiation was delivered within 90 ms to a cutaneous lesion in a patient with multi-resistant CD30^+^ T-cell lymphoma. The FLASH treatment resulted in only mild skin toxicity, which resolved more rapidly than those observed in a comparable lesion treated with conventional radiotherapy. Complete lesion remission was achieved at 6 months, with both modalities demonstrating similar acute and long-term (2-year) outcomes [11]. This landmark case provided initial evidence for the feasibility and safety of FLASH-RT within this dose range when targeting the skin. Building upon this pioneering experience, several prospective trials have been launched. The phase I IMPulse trial (NCT04986696) is currently evaluating dose-escalated electron FLASH-RT (22–34 Gy, 9 MeV) in patients with melanoma skin metastases, with primary endpoints focused on acute toxicity within 4 weeks and secondary endpoints evaluating late toxicity and local tumour response over 12 months [203]. Moreover, the University of Zurich also initiated a Phase I study (Flash-Skin-Ⅰ, NCT06549439) designed to evaluate the feasibility and safety of FLASH-RT specifically for melanoma skin metastases [204]. In parallel, a single-center randomized Phase II trial (LANCE, NCT05724875) has been launched to directly compare FLASH-RT with CONV-RT in patients with localized cutaneous squamous cell carcinoma or basal cell carcinoma [205]. Notably, Chinese researchers describe the first patient worldwide to receive fractionated FLASH-RT. After one-year follow-up, they showed that fractionated electron FLASH-RT is feasible and safe for the treatment of extramammary Paget disease of the scrotum [206]. These studies collectively signify a crucial translational step that systematically exploring the clinical potential of electron FLASH-RT across different skin malignancies. Besides electrons, proton FLASH-RT has also entered clinical investigation. The nonrandomized phase I study (FAST-01, NCT04592887) in patients with painful bone metastases of the extremities, demonstrated that proton FLASH-RT was clinically feasible and associated with minimal treatment-related adverse events [207]. These encouraging outcomes directly motivated the initiation of the subsequent FAST-02 clinical trial (NCT05524064), which extends proton FLASH-RT to thoracic bone metastases with a focus on pain relief, safety, and treatment efficiency. With an expected completion date in 2027, positive findings are anticipated to facilitate the broader clinical application of FLASH-RT to other indications [208].

**Table 4:** Clinical trials of FLASH-RT.

Radiation modality	Tumour type	Dose rate and Total dose	Endpoint	Stage & Status	Clinical trail identifier
Electrons (5.6 Mev)	Skin lymphoma	15 Gy in 90 ms	Skin toxicity and tumour response	Completed	/ [11]
Electrons (9 Mev)	Skin melanoma metastases	22, 24, 26, 28, 30, 32 and 34 Gy	Maximum tolerated dose; Hemorrhage; ulceration;	Phase I (Ongoing)	NCT04986696 [203]
Electrons (9 Mev)	Skin melanoma metastases	3 × 9 Gy; 2 × 9 Gy; 1 × 9 Gy	Dose limiting toxicity and dose accuracy;	Phase I Primary completed	NCT06549439 [204]
Electrons (9 Mev)	cSCC or BCC non-amenable to surgery	220–270 Gy/s; 22 Gy or 30 Gy in 5 fractions of 6 Gy	Grade≥3 skin toxicity and local control rate;	phase II (Ongoing)	NCT05724875 [205]
Proton	Painful bone metastasis(-es) in the extremities	≥40 Gy/s; 8 Gy in a single fraction	Patient time on treatment couch; device-related treatment delays; patient-reported pain scores; adverse event; analgesic use;	Completed	NCT04592887 [207]
Proton	Painful thoracic bone metastases	≥40 Gy/s; 8 Gy in a single fraction	Pain relief and safety; Any device issues as well as time on the treatment table;	Ongoing	NCT05524064 [208]
Electrons (9 Mev)	Paget disease of the scrotum	120 Gy/s; 40Gy in 5 fractions	Transient and long-term skin toxicity; Tumor respond	Completed	[206]

### 5.2 Challenges and unsolved problems in clinical translation of FLASH-RT

Despite the remarkable progress in clinical implementation, the translation of FLASH-RT from early-phase feasibility trials into routine clinical practice faces substantial hurdles. The path forward is constrained by unresolved biological mechanisms, technological limitations, and a paucity of robust clinical evidence. This section critically examines the key challenges that must be addressed to enable safe and effective clinical deployment of FLASH-RT.

#### 5.2.1 Uncertainties and fragmentation in biological mechanisms

Despite intensified research efforts, the biological mechanisms underlying the FLASH effect remain inadequately elucidated. Early studies focused predominantly on the oxygen depletion hypothesis, but this model is facing increasing challenges [209]. Recent years have witnessed an expanding repertoire of proposed mechanisms, including radical recombination, immune cell sparing, DNA integrity preservation, mitochondrial metabolism and vascular permeability modulation. New biological mechanisms continue to emerge. This phenomenon reflects both the increasing vitality of the field and exposes a fundamental problem: current research on FLASH-RT mechanisms is characterized by fragmentation, leading to an accumulation of hypotheses rather than theoretical construction. Each new study tends to emphasize a specific pathway or mediator, yet the interrelationships among these proposed hypotheses remain unexplored. For example, do DNA integrity preservation and immune cell retention represent parallel independent processes, or are they sequential events within a common cascade? These fragmented mechanisms may lead to the following problems:

**Inability to distinguish causal relationship.** The FLASH effect is not driven by a single molecular event but involves a systems-level response across multiple dimensions operating on extremely short timescales. This complexity urgently requires an integrative framework to delineate causal hierarchies: which are initiating events, which are amplification mechanisms, and which are tissue-specific modifying factors.

**Inability to explain anomalous phenomena.** The existence of late toxicity and inconsistent FLASH effects in certain tissues suggests that current protocols may be suboptimal. It remains to be determined whether these failures represent genuine biological limitations or are simply due to pulse structures and dose rates that fail to reach tissue-specific protective thresholds. Such uncertainty creates a significant hurdle for clinical translation.

**Inability to guide clinical practice.** In the absence of an integrative framework, we cannot answer the most fundamental questions: which patients should receive FLASH treatment? what dose and fractionation regimen should be selected? how can individual patient toxicity risk be predicted? Based on conflicting hypotheses, it is impossible to design clinical trials with adequate biological rationale.

Therefore, the field of FLASH research lacks a unified conceptual framework capable of integrating these diverse observations into a coherent biological principle. The construction of such a framework requires the collective effort of the entire community—sustained dialogue among experimental biologists, physicists, and clinicians.

#### 5.2.2 Physical and technical implementation barriers

The clinical translation of FLASH-RT is equally constrained by a series of physical and technical challenges that span the entire chain of treatment delivery, from beam production to dosimetric verification and treatment planning. These barriers are not merely engineering inconveniences; they strike at the heart of whether FLASH can be delivered with the precision, safety, and reproducibility required for routine clinical practice.

##### 5.2.2.1 The elusive definition of "FLASH conditions"

What physical parameters are necessary and sufficient to elicit the FLASH effect? The field relies on descriptive thresholds: average dose rates exceeding 40-100 Gy/s, total irradiation times below 200-500 ms, and fraction doses above 4-10 Gy as basic conditions [210]. However, these thresholds are phenomenological rather than mechanistic, and the relative importance of instantaneous versus average dose rate, pulse structure remains unresolved. Preclinical studies employ widely varying parameters (pulse dose rates from 10^2^ to 10^6^ Gy/s, pulse widths from microseconds to milliseconds), yet whether these are interchangeable remains unknown. In addition, the beam interruptions inevitable in clinical delivery (e.g., energy layer switching in proton therapy), may also compromise the FLASH effect [210]. Currently, the parameters selected for clinical trials, which often constrained by available technology rather than biological optimization, may or may not represent the conditions that maximize normal tissue protection. Consequently, a "FLASH effect" demonstrated with one experimental setup may not translate to another. Future experimental studies should systematically integrate tumor-site-specific clinical parameters to define the thresholds for the FLASH effect, including the minimum fraction dose and dose rate required for normal tissue sparing. Key priorities include establishing the maximum tolerable beam-off time to maintain the FLASH effect and determining whether a dose rate threshold also exists for tumor response. Furthermore, longitudinal studies are essential to comprehensively monitor and report the long-term effects of FLASH-RT.

##### 5.2.2.2 Technological limitations across beam modalities

Each radiation modality faces distinct barriers to clinical FLASH implementation. Electron FLASH, despite advanced clinical testing, is fundamentally limited to superficial targets by its shallow tissue penetration (4-12 MeV). To address this, the development of very high energy electron (VHEE) accelerators (100–250 MeV) has emerged as a primary technical pathway. By leveraging higher electron energies to enhance penetration and decreased spreading of the penumbra, these platforms aim to combine the UHDR capabilities of electrons with the dose profiles required for deep-seated tumours. While high-energy VHEE systems are technically feasible, their clinical adoption remains dependent on substantial advancements in accelerator engineering and shielding modifications, as well as the biological effect of VHEEs [90,211]. Proton FLASH offers the promise of deep tumor coverage combined with FLASH sparing. However, current delivery strategies face a fundamental trade-off between achievable dose rate and dose conformality. The highest instantaneous dose rates are most readily achieved using high-energy transmission beams (≈230–250 MeV), which bypass the Bragg peak and therefore may sacrifice the sharp distal fall-off that underpins the dosimetric superiority of proton therapy. To solve this, the next generation of proton delivery systems may focus on Bragg peak FLASH. This involves high-intensity synchrocyclotrons and advanced beam-scanning magnets capable of ultra-fast spot-scanning. Concurrent efforts in developing universal range shifters and target specific range compensators, which enables the combination of FLASH with SOBP from a mono-energetic beam, maintaining the sharp distal dose fall-off required for organs-at-risk(OAR) sparing and thereby ensuring the protection of normal tissues. [17,212]. Photon FLASH remains the least developed, as conventional linacs cannot achieve FLASH dose rates at depth due to inefficient bremsstrahlung conversion. To achieve X-ray FLASH-RT, a more powerful accelerator and an electron-to-photon conversion target that can tolerate instantaneous ultra-high dose rate are required. Emerging platforms such as the PHASER (Pluridirectional high-energy agile scanning electronic radiotherapy), superconducting electron accelerator from China Academy of Engineering Physics, and the compact linear accelerator developed by Tsinghua University, represent a transformative step toward achieving these requirements within standard clinical environments. In summary, all modalities ultimately require purpose-built accelerator designs that are only beginning to emerge from research laboratories. Achieving clinical translation will necessitate extensive biological validation and rigorous quality control to ensure the long-term stability and reliability of these accelerator systems.

##### 5.2.2.3 Dosimetric validation under UHDR conditions

The lack of established dosimetry protocols for ultra-high dose rate beams constitutes the primary technical hurdle for clinical implementation. In FLASH-RT, the real-time monitoring and active control of beam energy, dose rate, uniformity, and symmetry are critical to achieving the safe and controllable delivery of ultra-high dose rates. Conventional ionization chambers exhibit severe recombination effects at dose rates exceeding 10^5^ Gy/s, rendering their readings unreliable [213,214]. Therefore, the utilization of specialized ionization chambers designed specifically for FLASH applications is essential. Alternative detectors, such as Faraday cups, scintillators, radiochromic film and thermoluminescent dosimeter are currently under investigation, however, each possesses inherent limitations and there is no consensus on a reference dosimetry protocol [215]. Furthermore, the ultra-fast dose delivery characteristic of FLASH radiotherapy imposes significantly more stringent requirements on the precision of image guidance and respiratory gating compared to conventional radiotherapy, as even minor deviations carry a high risk of complete dosimetric target miss. Research is exploring the use of ultra-high instantaneous positron intensity generated during proton FLASH delivery to evaluate the potential of miniature PET scanners for FLASH beam imaging and dosimetry. Preliminary results have demonstrated the feasibility of using in-beam PET imaging to monitor FLASH proton beams [216]. Overall, preclinical studies lack unified dosimetric methods and standards, and it is necessary to reach a consensus as soon as possible and establish monitoring guidelines. The development of an online beam monitoring and quality assurance system compatible with UHDR is still in its early stages.

##### 5.2.2.4 Treatment planning: the missing dimension of dose rate

Current treatment planning systems optimize solely for physical dose distribution, but FLASH introduces a second critical dimension of dose rate distribution, that existing systems cannot handle. Incorporating dose rate constraints into inverse planning requires fundamental algorithmic development, as optimizers must simultaneously satisfy dose objectives and ensure normal tissues are irradiated under true FLASH conditions. Early explorations reveal inevitable trade-offs between FLASH coverage and conventional plan quality metrics, with acceptable balances yet undefined. Additionally, the spatial distribution of dose rate may not coincide with dose distribution (particularly in proton FLASH), raising unanswered questions about whether partial tumor coverage under FLASH conditions is clinically acceptable [217], highlighting an inherent trade-off between dose conformity, dose-rate eligibility, and clinical practicality that remains unresolved [218]. A further complicating factor is the lack of consensus on how dose rate should be defined and quantified. Various metrics, including mean dose rate (MDR), dose-averaged dose rate (DADR), and dose-threshold dose rate (DTDR), produce inconsistent estimates of FLASH coverage, thereby confounding both the optimization process and the clinical interpretation of treatment plans [218].

In summary, the clinical translation of FLASH-RT confronts multifaceted challenges spanning biological uncertainty, technological limitation, and evidence insufficiency. Addressing these challenges will require coordinated multidisciplinary efforts: preclinical experiments designed to yield phenomenological insights relevant to human application, technological innovations in beam delivery and dosimetry, and carefully designed clinical trials with sufficient follow-up to capture both acute and late effects. Only through such systematic investigation can the transformative potential of FLASH-RT be safely and effectively realized.

### 5.3 Safety considerations and risk mitigation

The existing clinical evidence base for FLASH-RT remains limited in both scope and depth. Published human data are confined to small, non-randomized trials in superficial targets (skin lesions) or palliative settings (bone metastases) with short follow-up durations. This is particularly concerning given that late effects including fibrosis, vascular damage, and secondary carcinogenesis are the primary determinants of therapeutic ratio in curative-intent radiotherapy. The enthusiasm of FLASH-RT should be tempered by appropriate caution regarding unforeseen toxicities. As editorial commentary has emphasized, "a rushed, premature clinical implementation with unforeseen toxicity would be harmful not only for the involved patients but for the entire field of FLASH". Finding the maximum safe dose that can be administered in a single irradiation in future preclinical experiments, continuously evaluating its late damage, and eliminating the risk of FLASH retaining effects in tumor are crucial for clinical translation of FLASH-RT in the future.

## 6 Future directions of FLASH-RT

### 6.1 Synergistic integration of FLASH-RT and immunotherapy

**Fig. 6:**
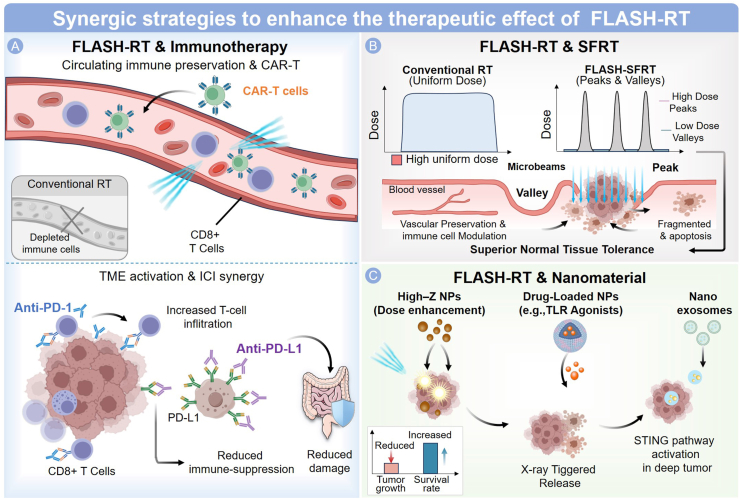
Synergistic therapeutic strategies combining ultra-high dose rate FLASH radiotherapy (FLASH-RT) with immunotherapy, spatially fractionated radiotherapy, and nanomaterials.

Radiotherapy has long been recognized as a promising partner for immunotherapy due to its predictable safety profile, widespread clinical availability, and capacity to induce immunogenic tumour cell death [219]. However, numerous studies combining radiotherapy with immune checkpoint inhibitors (ICIs) have failed to demonstrate meaningful synergy. This absence of synergistic effect might be attributed to suboptimal study design, choice of end points, especially the administration of conventional radiotherapy according to standard schedules and target volumes which has been reported to exacerbate immunosuppression in the tumour microenvironment [219]. The emergence of FLASH-RT has attracted interest in radioimmunotherapy, driven by its ability to mitigate normal tissue toxicity and modulate the TME while maintaining robust tumor control [220]. Preclinical studies suggest that combining FLASH-RT with immune checkpoint inhibitors (ICIs) may yield synergistic benefits (Fig. 6). For example, FLASH X-ray irradiation significantly minimized mouse enteritis by alleviating CD8^+^ T cell-mediated deleterious immune response compared with CONV-RT, but was as competent as CONV-RT in boosting the antitumor immune response and achieved equal tumour control in metastasis burdens when combined with anti-PD-L1 administration [48]. Similarly, FLASH-RT enhanced the efficacy of anti-PD-1 therapy in both immunotherapy sensitive or resistant ovarian cancer models, with decreased Treg to T effector ratio and increased intratumoral CD8^+^ T cell infiltration (Fig. 6). Most importantly, this finding demonstrated that combination of FLASH-RT with PD-1 inhibition does not increase gastrointestinal or hematological toxicities [87]. Beyond ICIs, FLASH-RT also has been showed to improve infiltration and activation of chimeric antigen receptor (CAR) T cells and sensitizes medulloblastoma to GD2 CAR-T cell therapy (Fig. 6) [180]. Despite these positive evidences, the translation of these synergistic effects requires a more critical appraisal of the underlying radiobiological mechanism. Although recent reports suggest that FLASH-RT can reduce the infiltration of immunosuppressive cells, such as Tregs and M2-type macrophages within the tumor microenvironment, these findings are largely constrained to specific dose and dose-rate configurations. The universality of these observations across diverse cancer types and varying dose-rate ranges remains unverified. A critical scientific concern is whether FLASH-RT might, under certain conditions, preserve these immunosuppressive populations and thereby attenuating its overall therapeutic benefit. Moreover, most of current evidence is based on long-term passaged cell-line derived xenograft (CDX) models, which often fail to recapitulate the complexity of the human TME. To address this, future investigations must prioritize patient-derived xenograft (PDX) models and utilize single-cell RNA sequencing and high-dimensional flow cytometry to delineate the dose-rate-dependent kinetics of diverse immune subsets. To date, clinical experience with the combination of FLASH-RT and immunotherapy remains extremely limited, and no such strategies have yet entered routine clinical testing. Establishing optimal sequencing, dosing regimens, and patient selection criteria, alongside a thorough assessment of long-term immune-related toxicities, will be essential for the successful clinical translation of these synergistic effects.

### 6.2 Combining FLASH-RT with spatially fractionated radiation therapy(SFRT)

The concept of spatially modulating the dose in RT is enjoying a huge revival in recent years with the development of radiotherapy equipment and techniques. Spatially fractionated radiation therapy (SFRT) represents another disruptive approach that challenges traditional radiotherapy paradigms [221]. In SFRT, the dose is spatially modulated to create highly non-uniform dose distributions composed of alternating high-dose “peaks” and low-dose “valleys”, fundamentally contrasting with the flat dose profiles used in conventional radiotherapy. Depending on beamlet size and geometry, SFRT can be classified into GRID-RT and lattice RT (LRT) use centimeter-scale beamlets, minibeam RT (MBRT) and microbeam RT (MRT) work with beamlets in the range of hundreds and tens of micrometers, respectively [222]. The high spatial dose modulation in SFRT activates distinct radiobiological effect that demonstrated remarkable normal tissue tolerance and therapeutic efficacy, especially in bulky and radioresistant tumours [221]. Although its biological mechanisms are not yet fully understood, currently studies has been showed that the proposed contributors include differential vascular effect [223], immune and inflammatory modulation [224], and bystander effects [221].

Both SFRT and FLASH-RT independently offer enhanced tumor control and improved normal tissue sparing, thus the synergistic potential of combining these approaches may provide a novel paradigm for enhancing the protection of healthy tissues and improve tumor control [222,225]. Indeed, the combination of FLASH-RT and SFRT has been successfully implemented in synchrotron based MRT and MBRT platforms (Fig. 6) [[226], [227], [228]]. In a recent rat lung study, FLASH microbeam radiotherapy exhibited superior normal tissue tolerance compared with both FLASH broad-beam irradiation, with markedly reduced fibrotic lesions, inflammation, bronchial and vascular damage [229]. These findings suggest that spatial dose heterogeneity may further potentiate the normal tissue–sparing effects of FLASH-RT, opening the door to investigations of the combining efficacy of FLASH-RT and SFRT. However, in another *in vivo* study of total body irradiation with synchrotron MRT at 291 Gy/s, UHDR broad beam RT at 39 Gy/s, and conventional RT at 0.05 Gy/s, a normal tissue spare effect could not detected even without spatial fractionation, underscoring the critical influence of experimental controls and parameter selection [62]. In all, due to the fact that both SFRT and FLASH-RT have already proven to offer a remarkable reduction of normal tissue toxicities while providing similar or even superior tumour control, combination of SFRT and FLASH-RT could have the potential to further increase therapeutic index and benefit the treatment of radioresistant tumours. However, direct evidence of synergistic biological effects between FLASH-RT and SFRT remains limited. A fundamental challenge lies in the complex spatiotemporal orchestration of delivery: does a synergistic window exist between the temporal FLASH parameters (pulse structure, instantaneous dose rate) and the spatial SFRT parameters (beamlet geometry, peak-to-valley dose ratio (PVDR), and spatial duty cycle)? These parameters may not independent; rather, the rapid oxygen depletion characteristic of FLASH-RT might be fundamentally altered by the localized dose gradients of SFRT. If the spatial valleys in SFRT prevent the FLASH-induced radical kinetics from reaching the threshold required for normal tissue sparing, the anticipated synergy could be compromised. Addressing this challenge requires extensive experimental validation. It is essential to construct a spatiotemporal dose-response landscape that correlates FLASH temporal parameters with SFRT spatial configurations, thereby identifying the specific conditions where FLASH temporal dynamics and SFRT spatial heterogeneity intersect to maximize therapeutic gain.

### 6.3 Integration of FLASH-RT with nanomaterials-based therapeutic strategies

Nanomedicine employs organic nanomaterials as carriers and has emerged as a powerful platform for targeted cancer therapy. Nanoparticle (NP)-based therapies, including photothermal therapy (PTT), photodynamic therapy (PDT), sonodynamic therapy (SDT), and chemo-dynamic therapy (CDT), have demonstrated considerable potential in addressing cancer recurrence and metastasis. In recent decades, NP-based radiosensitizers have emerged as promising agents showing feasibility and safety in enhancing radiotherapy efficiency. As summarized by Paul et al., the interaction between X-ray irradiation and NPs can be categorized into three primary mechanisms according to its chemical nature:(i) high atomic number (Z) NPs, which increase secondary electron emission and thus improve conventional radiotherapy efficacy through enhancing photoelectric and Compton effects; (ii) X-ray triggered drug-releasing NPs, which enable controlled payload release upon irradiation; (iii) Self-lighting photodynamic NPs, once irradiated by X-rays, the scintillator core emits a visible light and activates a photosensitizer that generates singlet oxygen (^1^O_2_) for tumour destruction [230]; The radiosensitizing effect of NPs is influenced by both the energy of the incident photon beam and the specific irradiation setup [230]. Recently, several innovative nanoplatforms have been developed to synergize with FLASH-RT. Dong et al. designed a a radiopaque, radiation-responsive hydrogel to deliver the Toll-like receptor (TLR) agonist imiquimod (IMQ) in conjunction with FLASH-RT, achieving enhanced tumour control and elevated intratumoral cytokine levels in melanoma models [231]. In another study, a biomimetic nanoplatform (TAFL) co-loading aggregation-induced emission photothermal agents (TPE-BBT) and anti-cancer drugs aspirin, has been designed to clear CSCs. *In vivo* results demonstrated that TAFL-mediated reduction of the CSCs population significantly inhibited tumour recurrence and metastasis after FLASH-RT therapy [232]. Further advancing precise immunotherapy, researchers utilized nanoexosomes loaded within porous microneedles for precise delivery and accumulation of the STING agonist MSA-2 (MEM) to deep residual tumours, which promoted STING pathway activation upon exposure FLASH-RT, eliciting a potent immune response that effectively prevented recurrence while minimizing systemic toxicity (Fig. 6) [233]. These emerging strategies highlight the potential of nanotechnology to further enhance the therapeutic window of FLASH-RT by enabling spatially and temporally precise immune and radiosensitizing interventions.

## 7 Conclusions

The advent of FLASH radiotherapy marks a pivotal moment in oncologic therapy, promising to decouple tumor eradication from normal tissue toxicity—a long standing goal in radiation oncology. Extensive evidence consistently demonstrated that ultra-high dose rate irradiation confers robust protection to radiosensitive organs such as the brain, lung, and intestines, while preserving, and in some contexts enhancing, antitumor efficacy. Mechanistically, the "FLASH effect" dose not arise form a single pathway but from a confluence of radiobiological events, including transient physicochemical conditions that alter radical chemistry, differential induction of DNA damage and repair, and profound modulations of inflammatory and immune pathways. Notably, the capacity of FLASH-RT to spare circulating lymphocytes and activate favorable immune responses within tumours positions making it is a compelling partner for synergistic combination with immunotherapy, potentially overcoming key limitations of conventional radio-immunotherapy regimens. The journey toward routine clinical implementation, however, is underscored by critical challenges. A precise understanding of the parameter space encompassing dose rate thresholds, fractionation schedules, and particle-type dependencies is essential. Furthermore, the development of reliable, clinically adaptable delivery systems for photons and protons is imperative to extend FLASH benefits beyond superficial tumours. Future research must prioritize the validation of mechanistic models in human-relevant contexts, the execution of robust comparative clinical trials, and the innovative integration of FLASH with adjuvant therapies like targeted nanocarriers and spatially fractionated techniques.

In conclusion, FLASH radiotherapy transcends a mere technical increment in dose rate; it embodies a transformative approach with the potential to fundamentally widen the therapeutic window. By mitigating dose-limiting toxicities, it may enable the safe delivery of more aggressive, even curative, radiation doses and unlock potent combinatorial strategies. As technological and biological insights converge, FLASH-RT is poised to transition from a compelling laboratory phenomenon to a cornerstone of next-generation precision oncology, ultimately aiming to improve survival and quality of life for cancer patients worldwide.

**Table:** List of abbreviations

FLASH radiotherapy	FLASH-RT
Radiotherapy	RT
Image-guided radiotherapy	IGRT
Adaptive radiotherapy	ART
Ultra-high dose rate	UHDR
Conventional radiotherapy	CONV-RT
Radiation dermatitis	RD
Dose-modifying factor	DMF
Cranial radiation therapy	CRT
Radiation-induced brain injury	RIBI
Reactive oxygen species	ROS
Blood-brain barrier	BBB
Radiation-induced lung injury	RILI
Total abdominal irradiation	TAI
Mean dose rates	MDR
Dose per pulse	DPP
Instantaneous dose rate	IDR
Long-term potentiation	LTP
Stereotactic body radiotherapy	SBRT
Very high energy electrons	VHEE
Kilovoltage	KV
Megavoltage	MV
Pencil beam scanning	PBS
Spread-out Bragg peaks	SOBP
Relative biological effectiveness	RBE
Radiolytic Oxygen depletion	ROD
Oxygen enhancement ratio	OER
Mitochondrial DNA	mtDNA
Single-strand breaks	SSB
Cancer stem cells	CSC
Damage-associated molecular pattern	DAMP
Epithelial–mesenchymal transition	EMT
Tumor immune microenvironment	TIMI
Spatially fractionated radiation therapy	SFRT
Low-dose radiotherapy	LDRT
Neutrophil extracellular trap	NET
Immune checkpoint inhibitors	ICI
Chimeric antigen receptor	CAR
Lattice RT	LRT
Minibeam RT	MBRT
Microbeam RT	MRT
Photothermal therapy	PTT
Nanoparticle	NP
Photodynamic therapy	PDT
Sonodynamic therapy	SDT
Chemo-dynamic therapy	CDT

## Ethics approval

Not applicable.

## Consent for publication

Not applicable.

## Availability of data and materials

Not applicable.

## Funding

This work was supported by grants from the Project [ZR2025QC1485] supported by 10.13039/501100007129Shandong Provincial Natural Science Foundation; Project [12505409] supported by 10.13039/501100001809National Natural Science Foundation of China; Project [ZR2025QC2150Z] supported by 10.13039/501100007129Shandong Provincial Natural Science Foundation; The Young Scientists Fund of the 10.13039/501100001809National Natural Science Foundation of China [No. 12405395]; the 10.13039/100012556Youth Science and Technology Foundation of Gansu Province [No. 24JRRA998].

## CRediT authorship contribution statement

**Zhihui Dou:** Writing – original draft. **Huiwen Lei:** Writing – review & editing. **Jingfen Yang:** Writing – review & editing. **MengYao Li:** Writing – review & editing. **Shuting Wang:** Writing – review & editing. **Xingting Bao:** Writing – original draft. **Li Ma:** Writing – review & editing. **Jinjiang Li:** Writing – review & editing. **Yong Yin:** Conceptualization, Writing – review & editing. **Tianyuan Dai:** Conceptualization, Writing – review & editing.

## Declaration of competing interest

The authors declare that they have no known competing financial interests or personal relationships that could have appeared to influence the work reported in this paper.

## Data Availability

No data was used for the research described in the article.
